# Parathyrsoidins A–D, Four New Sesquiterpenoids from the Soft Coral *Paralemnalia thyrsoides*

**DOI:** 10.3390/md11072501

**Published:** 2013-07-12

**Authors:** Yen-Ju Tseng, Yu-Sheng Lee, Shang-Kwei Wang, Jyh-Horng Sheu, Chang-Yih Duh

**Affiliations:** 1 Department of Marine Biotechnology and Resources, National Sun Yat-sen University, Kaohsiung 804, Taiwan; E-Mails: pit0424@yahoo.com.tw (Y.-J.T.); m995020028@student.nsysu.edu.tw (Y.-S.L.); sheu@mail.nsysu.edu.tw (J.-H.S.); 2 Department of Microbiology, Kaohsiung Medical University, Kaohsiung 807, Taiwan; 3 Asia-Pacific Ocean Research Center, National Sun Yat-sen University, Kaohsiung 804, Taiwan

**Keywords:** softcoral, *Paralemnalia thyrsoides*, parathyrsoidins A–D, cytotoxicity

## Abstract

Four new nardosinane-type sesquiterpenoids, parathyrsoidins A–D (**1**–**4**) were isolated from the soft coral *Paralemnalia thyrsoides*. The structures of parathyrsoidins A–D (**1**–**4**) were determined by extensive spectral analysis and their cytotoxicity against selected cancer cell lines as well as antiviral activity against human cytomegalovirus (HCMV) were evaluated *in vitro*.

## 1. Introduction

**Figure 1:**
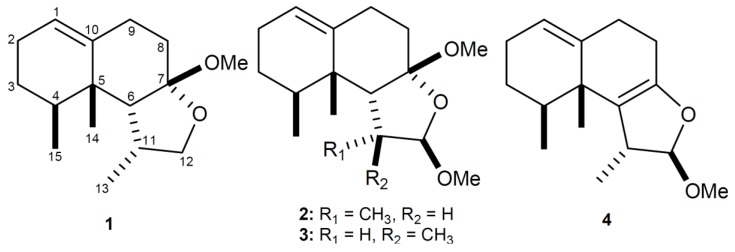
Structures of Metabolites **1**–**4**.

Soft corals belonging to the genus *Paralemnalia* have been proved to be a rich source of sesquiterpenoids [1,2,3,4,5,6,7,8,9,10,11,12,13]. Our recent study of the chemical constituents of the soft coral *P. thyrsoides* has yielded a cytotoxic bisnorsesquiterpenoid, paralemnolide A [13]. Continuing chemical investigation of the same collection led to the isolation of four new nardosinanes, parathyrsoidins A–D (**1**–**4**) (Figure 1). The relative structures of these metabolites were established by extensive spectroscopic analysis. The cytotoxicity of **1**–**4** against P-388 (mouse lymphocytic leukemia), HT-29 (human colon adenocarcinoma), and A-549 (human lung carcinoma) cancer cell lines as well as antiviral activity against human cytomegalovirus (HCMV) were evaluated *in vitro*.

## 2. Results and Discussion

**Figure 2:**
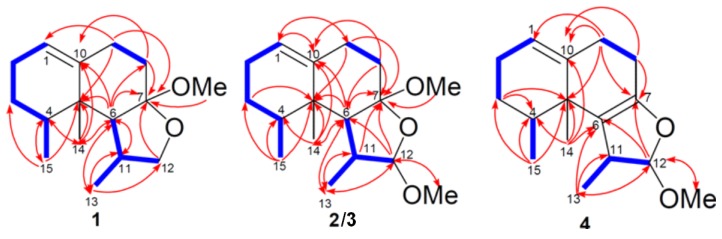
Selected ^1^H−^1^H COSY (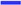) and HMBC (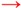) correlations of **1**–**4**.

**Figure 3:**
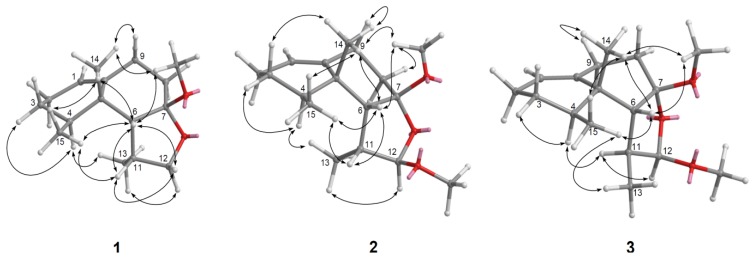
Key NOESY Correlations for **1**–**3**.

Parathyrsoidin A (**1**) was isolated as an amorphous solid. Its molecular formula, C_16_H_26_O_2_, was established by HRESIMS (*m/z* 273.1831, [M + Na]^+^), implying four degrees of unsaturation. The ^13^C NMR and DEPT (Table 1) spectroscopic data showed signals of four methyls (including one oxymethyl), five sp^3^ methylenes (including one oxymethylene), three sp^3^ methines, one sp^2^ methine, two sp^3^ (including one quaternary sp^3^ dioxycarbon) and one sp^2^ quaternary carbons. The NMR signals (Table 1) observed at δ_C_ 120.7 (CH) and 141.9 (C), δ_H_ 5.28 brd, *J* = 5.2 Hz showed the presence of one trisubstituted double bond. The above data accounted for one of the four degrees of unsaturation, thus, the tricyclic structure of **1** was revealed. In the ^1^H–^1^H COSY spectrum, it was possible to identify three different structural units, which were assembled with the assistance of an HMBC experiment. Key HMBC correlations of H-4 to C-14 and C-15; H-6 to C-5, C-7, C-8, C-10, C-11, C-13 and C-14; H_2_-8 to C-7 and C-10; H_2_-9 to C-1, C-5 and C-7; H-11 to C-6 and C-13; H_2_-12 to C-7; H_3_-13 to C-6, C-11 and C-12; H_3_-14 to C-4, C-5, C-6 and C-10; H_3_-15 to C-3, C-4, C-5 permitted the establishment of the nardosinane-type skeleton of **1** (Figure 2). Detailed analysis of 2D NMR correlations (Figure 2) and comparison of the ^1^H NMR data (in CDCl_3_) **1** (Supplementary Materials) with those of 2-deoxy-7-*O*-methyllemnacarnol [14] suggested that **1** is a closely related isomer of 2-deoxy-7-*O*-methyllemnacarnol. The relative configuration of **1** was proposed on the basis of key NOE correlations (Figure 3). The NOE correlations between H_3_-14/H-6, H_3_-14/H_3_-15, H_3_-14/OMe-7, and H-6/H-11 showed that H-6, H-11, H_3_-14, H_3_-15, and OMe-7 are located on the β face, whereas H-4 and H_3_-13 are oriented toward the opposite face. According to the aforementioned observations, the structure of parathyrsoidin A (**1**) was revealed to be the C-11 epimer of 2-deoxy-7-*O*-methyllemnacarnol. In order to rule out the possibility of **1** being an isolation artifact, a sample of 2-deoxylemnacarnol [5] obtained from Prof. Sheu laboratory was kept at room temperature for three day in the presence of RP-18 gel in MeOH. However, the formation of 2-deoxy-7-*O*-methyllemnacarnol was not observed.

**Table 1:** ^1^H and ^13^C NMR spectroscopic data for compounds **1** and **4**.

Position	1		4	
	δ_H_^a^	δ_C_^b^	δ_H_^a^	δ_C_^b^
1	5.28 brd (5.2) ^c^	120.7, CH ^d^	5.29 brs	120.9, CH
2	1.89 m, 1.94 m	26.0, CH_2_	1.82 m, 1.88 m	25.7, CH_2_
3α 3β	1.28 m 1.42 qd (12.0, 6.0)	27.2, CH_2_	1.28 m 1.37 qd (13.2, 6.4)	28.9, CH_2_
4	1.78 dqd (12.0, 6.8, 3.2)	35.0, CH	1.71 dqd (13.2, 6.4, 3.6)	37.8, CH
5		38.6, qC		39.2, qC
6	2.32 d (6.0)	52.7, CH		118.8, qC
7		107.5, qC		150.1, qC
8α 8β	1.81 td (13.6, 5.2) 2.31 dt (13.6, 5.2,)	35.3, CH_2_	2.19 m	26.4, CH_2_
9α 9β	1.98 ddd (13.6, 5.2, 1.2) 2.52 tdq (13.6, 5.2, 2.4)	29.2, CH_2_	1.95 ddd (12.8, 6.0, 1.2) 2.45 m	29.9, CH_2_
10		141.9, qC		142.7, qC
11	1.98 qdd (7.2, 6.0, 4.0)	36.7, CH	2.99 qd (7.2, 1.2)	46.4, CH
12α 12β	3.40 d (8.0) 3.78 dd (8.0, 4.0)	74.1, CH_2_	4.82 d (1.2)	112.5, CH
13	1.03 d (7.2)	15.9, CH_3_	1.12 d (7.2)	19.6, CH_3_
14	1.19 s	21.8, CH_3_	1.10 s	21.5, CH_3_
15	0.80 d (6.8)	16.4, CH_3_	0.95 d (6.4)	18.9, CH_3_
OMe-7 OMe-12	3.26 s	48.5, CH_3_		
			3.28 s	54.8, CH_3_

^a^ Spectra recorded at 400 MHz in C_6_D_6_. ^b^ Spectra recorded at 100 MHz in C_6_D_6_. ^c^ *J* values (in Hz) are in parentheses. ^d^ Multiplicities are deduced by HSQC and DEPT experiments.

Parathyrsoidin B (**2**) had the molecular formula C_17_H_28_O_3_, 30 mass units higher than that of **1**. Comparison of the ^1^H and ^13^C NMR data (Table 2) of compounds **1** and **2** showed that both compounds should have similar structures. C-12 of **2** showed signal at downfield δ_C_ 109.0, CH relative to the corresponding signal of **1** (δ_C_ 74.1, CH_2_), implying the presence of an oxymethyl at C-12 of **2**. In the 2D NMR spectra, including ^1^H–^1^H COSY and HMBC (Figure 2), three segregate consecutive proton spin systems, H-1 to H-4, H-4 to H_3_-15, H_2_-8 to H_2_-9, and H-11 to H-6, H-12 and H_3_-13, were found in the ^1^H–^1^H COSY spectrum. Detailed analysis of HMBC correlations (Figure 2) and comparison of ^1^H NMR data (in CDCl_3_) of **2** (Supplementary Materials) with those of 2-deoxy-12α-methoxy-7-*O*-methyllemnacarnol [14] revealed that they were closely related isomers. The relative configuration of **2** was determined by the analysis of NOE correlations, as shown in Figure 3. NOE correlations between H_3_-14/H-6, H_3_-14/H_3_-15, H_3_-14/OMe-7, and H-6/H-11 positioned the β-orientation of the aforementioned protons. Furthermore H_3_-13 (δ 1.00, d, *J* = 6.8 Hz) was found to show NOE interactions with H-4 (δ 1.74, dqd, *J* = 12.0, 6.8, 3.2 Hz) and H-12 (δ 4.37, s), suggesting the α-orientation of H-4, H-12 and H_3_-13. On the basis of the above findings (Figure 3), the relative structure of parathyrsoidin B (**2**) was determined as the C-11 epimer of 2-deoxy-12β-methoxy-7-*O*-methyllemnacarnol.

Parathyrsoidin C (**3**) possessed the same molecular formula (C_17_H_28_O_3_) as that of **2**, as established by HRESIMS and ^13^C NMR spectroscopic data. Further comparison of the ^1^H NMR (in CDCl_3_) and other spectral data among **3**, **2** (Table 2, Figure 2, and Supplementary Materials) and 2-deoxy-12α-methoxy-7-*O*-methyllemnacarnol [14] disclosed that three metabolites were closely related isomers. The significant coupling constant (*J*_H-11,12_ and *J*_H-11,6_) differences between **3** and **2** demonstrated that the configuration at C-11 for **3** differed from that of **2**. NOESY correlations between H_3_-15/H-6, H_3_-15/H_3_-13, H_3_-15/H_3_-14 suggested the β-orientations of H_3_-13, H_3_-14, H_3_-15, and H-6. Furthermore, H-11 was found to show NOESY correlations with both H-12 and H-4, suggesting the α-orientations of H-12, H-11, and H-4. Therefore, **3** was elucidated as the C-11 epimer of **2**.

**Figure 4:**
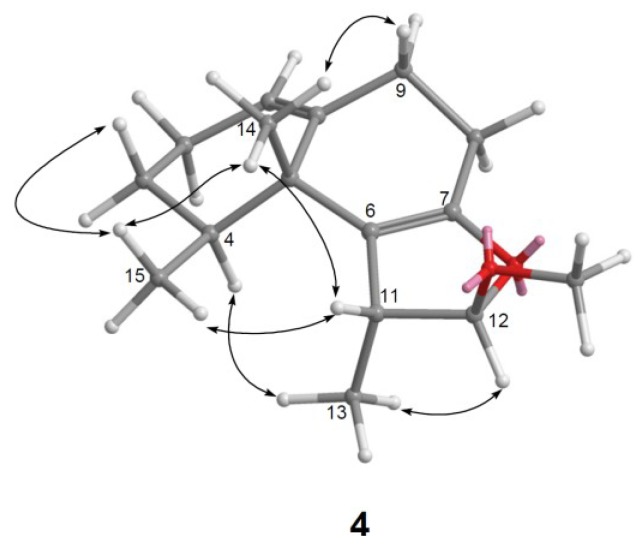
Key NOESY correlations for **4**.

Parathyrsoidin D (**4**) was also isolated as an amorphous solid and had the molecular formula C_16_H_24_O_2_, as determined by HRESIMS (C_16_H_24_O_2_ + Na, *m/z* found 271.1673, calculated 271.1674) indicating five degrees of unsaturation. Comparison of the NMR data (Table 1, Table 2) of compounds **2** and **4** showed both compounds should have similar structures. C-6 and C-7 of **4** showed signals at downfield δ_C_ 118.8, qC and 150.1, qC relative to the corresponding signals of **2** (δ_C_ 53.6, CH and 111.9, qC), implying the presence of a tetrasubstituted double bond at C-6 and C-7 of **4**. The planar structure of **4** was also confirmed by the ^1^H–^1^H COSY and HMBC correlations (Figure 2). The relative configuration of **4** was determined by NOE correlations. NOE correlations between H_3_-14/H-11 and H_3_-14/H_3_-15 positioned the β-orientation of the aforementioned protons, whereas H_3_-13, H-4, and H-12 are oriented toward the opposite surface (Figure 4).

**Table 2:** ^1^H and ^13^C NMR spectroscopic data for compounds **2** and **3**.

Position	2		3	
	δ_H_^a^	δ_C_^b^	δ_H_^a^	δ_C_^b^
1	5.56 t (2.8) ^c^	120.8, CH	5.42 brs	122.7, CH ^d^
2	1.84 m, 1.90 m	26.1, CH_2_	1.90 m, 1.96 m	25.7, CH_2_
3α 3β	1.23 m 1.35 qd (12.0, 6.0)	27.1, CH_2_	1.29 m 1.47 qd (13.2, 7.2)	27.1, CH_2_
4	1.74 dqd (12.0, 6.8, 3.2)	35.2, CH	1.71 dqd (13.2, 6.8, 3.2)	35.3, CH
5		38.4, qC		40.3, qC
6	2.86 dd (6.8, 2.4)	45.8, CH	2.40 d (10.8)	53.6, CH
7		109.6, qC		111.9, qC
8α 8β	1.81 td (14.4, 5.2) 2.38 ddt (14.4, 5.6, 2.4)	38.0, CH_2_	1.95 m	32.4, CH_2_
9α 9β	1.92 ddd (14.4,5.2, 2.4) 2.62 tdq (14.4, 5.6, 2.4)	29.7, CH_2_	2.33 m 2.21 tdq (14.0, 5.6, 1.6)	27.4, CH_2_
10		142.0, qC		140.2, qC
11	2.31 quin (6.8)	42.0, CH	1.83 dqd (10.8, 7.2, 5.6)	42.1, CH
12	4.37 s	109.0, CH	4.54 d (5.6)	105.9, CH
13	1.00 d (6.8)	14.2, CH_3_	1.15 d (7.2)	15.6, CH_3_
14	1.17 s	21.0, CH_3_	1.16 s	21.9, CH_3_
15	0.83 d (6.8)	16.2, CH_3_	0.75 d (6.8)	16.3, CH_3_
OMe-7 OMe-12	3.33 s 3.23 s	48.7, CH_3_ 54.3, CH_3_	3.32 s 3.27 s	48.9, CH_3_ 54.5, CH_3_

^a^ Spectra recorded at 400 MHz in C_6_D_6_. ^b^ Spectra recorded at 100 MHz in C_6_D_6_. ^c^ *J* values (in Hz) are in parentheses. ^d^ Multiplicities are deduced by HSQC and DEPT experiments.

Cytotoxicity of compounds **1**–**4** against the proliferation of a limited panel of cancer cell lines, including P-388, A549, and HT-29, were evaluated (Table 3). Metabolites **1**–**4** displayed moderate cytotoxicity against P-388, with ED_50_ of 7.95, 13.2, 3.63 and 2.32 μM, respectively. Compounds **1**–**4** were also examined for antiviral activity against HCMV using a human embryonic lung (HEL) cell line. Parathyrsoidins A–D (**1**–**4**) did not show anti-HCMV activity.

**Table 3:** Cytotoxicity of compounds **1**–**4** (ED_50_ μM).

Compound	A-549	HT-29	P-388
1	>20	>20	7.95
2	>20	>20	13.2
3	>20	>20	3.63
4 Mithramycin	>20 0.17	>20 0.19	2.32 0.14

## 3. Experimental Section

### 3.1. General Experimental Procedures

Optical rotations were measured on a JASCO P1020 digital polarimeter. IR spectra were recorded on a JASCO FT/IR4100 infrared spectrophotometer. The NMR spectra were recorded on a Varian MR 400 FT-NMR at 400 MHz for ^1^H (δ_H_ 7.16) and 100 MHz for ^13^C (δ_C_ 128.4), in C_6_D_6_ using solvent peak as internal standard. LRMS and HRMS were obtained by ESI on a Bruker APEX ÉÉ mass spectrometer. Silica gel 60 (Merck, 230–400 mesh) and LiChroprep RP-18 (Merck, 40–63 μm) were used for column chromatography. Precoated silica gel plates (Merck, Kieselgel 60 F_254_, 0.25 mm) and precoated RP-18 F_254s_ plates (Merck) were used for TLC analysis. High-performance liquid chromatography was carried out using a Hitachi L-7100 pump equipped with a Hitachi L-7400 UV detector at 220 nm together with a semi-preparative reversed-phase column (Merck, Hibar LiChrospher RP-18e, 5 μm, 250 × 25 mm).

### 3.2. Animal Material

The octocoral *P. thyrsoides* was collected by hand using scuba at the Sansiantai, Taitong County, Taiwan, in July 2008, at a depth of 6 m. This soft coral was identified by Prof. Chang-Fong Dai, Institute of Oceanography, National Taiwan University. A voucher specimen (SST-07) was deposited in the Department of Marine Biotechnology and Resources, National Sun Yat-sen University.

### 3.3. Extraction and Separation

The frozen soft coral was chopped into small pieces and extracted with acetone in a percolator at room temperature. The acetone extract of *P. thyrsoides* was concentrated to a brown gum, which was partitioned with EtOAc and H_2_O. The EtOAc-soluble residue (20 g) was subjected to Si 60 CC using *n*-hexane–EtOAc mixtures of increasing polarity for elution. Fraction 14, eluted with *n*-hexane–EtOAc (2:1), was purified by reverse-phase HPLC (MeOH–H_2_O, 85:15) to afford **1** (3.0 mg) and **2** (8.0 mg). Fraction 15, eluted with *n*-hexane–EtOAc (1:1), was purified by reverse-phase HPLC (MeOH–H_2_O, 85:15) to afford **3** (6.0 mg) and **4** (1.0 mg).

Parathyrsoidin A (**1**): amorphous solid; [α]^25^_D_ = −21 (*c* 0.8, CHCl_3_); IR (neat) ν_max_ 2962, 2857, 1460, 1338, 1250, 1098, 854 cm^−2^; ^1^H and ^13^C NMR data, see Table 1; ESIMS *m*/*z* 273 [M + Na]^+^; HRESIMS *m*/*z* 273.1831 (calcd for C_16_H_26_O_2_Na, 273.1830).

Parathyrsoidin B (**2**): amorphous solid; [α]^25^_D_ = −62 (*c* 2.0, CHCl_3_); IR (neat) ν_max_ 2991, 2960, 1385, 1274, 1100, 855 cm^−1^; ^1^H and ^13^C NMR data, see Table 1; ESIMS *m*/*z* 303 [M + Na]^+^; HRESIMS *m*/*z* 303.1937 (calcd for C_17_H_28_O_3_Na, 303.1936).

Parathyrsoidin C (**3**): colorless oil; [α]^25^_D_ = −42 (*c* 1.0, CHCl_3_); IR (neat) ν_max_ 2929, 2831, 1385, 1108, 864 cm^−1^; ^1^H and ^13^C NMR data, see Table 2; ESIMS *m*/*z* 303 [M + Na]^+^; HRESIMS *m*/*z* 303.1934 (calcd for C_17_H_28_O_3_Na, 303.1936).

Parathyrsoidin D (**4**): colorless oil; [α]^25^_D_ = −10 (*c* 0.1, CHCl_3_); IR (neat) ν_max_ 2957, 1684, 1653, 1377, 1100, 823 cm^−1^; ^1^H and ^13^C NMR data, see Table 2; ESIMS *m*/*z* 271 [M + Na]^+^; HRESIMS *m*/*z* 271.1673 (calcd for C_16_H_24_O_2_Na, 271.1674).

### 3.4. Cytotoxicity Testing

Cytotoxicity was determined on P-388 (mouse lymphocytic leukemia), HT-29 (human colon adenocarcinoma), and A-549 (human lung epithelial carcinoma) tumor cells using a modification of the MTT colorimetric method according to a previously described procedure [15,16]. The provision of the P-388 cell line was supported by J.M. Pezzuto, formerly of the Department of Medicinal Chemistry and Pharmacognosy, University of Illinois at Chicago. HT-29 and A-549 cell lines were purchased from the American Type Culture Collection. To measure the cytotoxic activities of tested compounds, five concentrations with three replications were performed on each cell line. Mithramycin was used as a positive control.

### 3.5. Anti-HCMV Assay

To determine the effects of natural products upon HCMV cytopathic effect (CPE), confluent human embryonic lung (HEL) cells grown in 24-well plates were incubated for 1 h in the presence or absence of various concentrations of tested natural products with three replications. Ganciclovir was used as a positive control. Then, cells were infected with HCMV at an input of 1000 pfu (plaque forming units) per well of a 24-well dish. Antiviral activity was expressed as IC_50_ (50% inhibitory concentration), or compound concentration required to reduce virus induced CPE by 50% after 7 days as compared with the untreated control. To monitor the cell growth upon treating with natural products, an MTT-colorimetric assay was employed [17].

## 4. Conclusions

In our continuing investigation of soft coral *P. thyrsoides* collected at San-Hsian-Tai (Taitong County, Taiwan) has led to the isolation of four new nardosinane-type sesquiterpenoids, parathyrsoidins A–D (**1**–**4**) exhibiting cytotoxicity against P-388 cell line with ED_50_ of 7.95, 13.2, 3.63 and 2.32 μg/mL, respectively. 13α-Methylated nardosinane-type metabolites have been found in soft corals *Lemnalia africana* and *Nephthea elongata* [2,4].

## Supplementary Files

Supplementary File 1 Supplementary Materials (PDF, 1319 KB)
