# Supplementary material for: Parathyrsoidins A–D, Four New Sesquiterpenoids from the Soft Coral Paralemnalia thyrsoides
Source: Mar Drugs. 2013 Jul 12;11(7):2501–9. doi: 10.3390/md11072501 (PMC3736437; doi:10.3390/md11072501)

Supplementary Materials

Table of Contents

Figure S1. ^{1}H NMR spectrum (300 MHz) of parathyrsoidin A (**1**) in $CDCl_3$. 2
Figure S2. ^{1}H NMR spectrum (400 MHz) of parathyrsoidin A (**1**) in C_6D_6. 3
Figure S3. ^{13}C NMR spectrum (400 MHz) of parathyrsoidin A (**1**) in C_6D_6. 4
Figure S4. COSY spectrum (400 MHz) of parathyrsoidin A (**1**) in C_6D_6. 5
Figure S5. HSQC spectrum (400 MHz) of parathyrsoidin A (**1**) in C_6D_6. 6
Figure S6. HMBC spectrum (400 MHz) of parathyrsoidin A (**1**) in C_6D_6. 7
Figure S7. NOESY spectrum (400 MHz) of parathyrsoidin A (**1**) in C_6D_6. 8
Figure S8. ^{1}H NMR spectrum (400 MHz) of parathyrsoidin B (**2**) in $CDCl_3$. 9
Figure S9. ^{1}H NMR spectrum (400 MHz) of parathyrsoidin B (**2**) in C_6D_6. 10
Figure S10. ^{13}C NMR spectrum (400 MHz) of parathyrsoidin B (**2**) in C_6D_6. 11
Figure S11. COSY spectrum (400 MHz) of parathyrsoidin B (**2**) in C_6D_6. 12
Figure S12. HSQC spectrum (400 MHz) of parathyrsoidin B (**2**) in C_6D_6. 13
Figure S13. HMBC spectrum (400 MHz) of parathyrsoidin B (**2**) in C_6D_6. 14
Figure S14. NOESY spectrum (400 MHz) of parathyrsoidin B (**2**) in C_6D_6. 15
Figure S15. ^{1}H NMR spectrum (400 MHz) of parathyrsoidin C (**3**) in $CDCl_3$. 16
Figure S16. ^{1}H NMR spectrum (400 MHz) of parathyrsoidin C (**3**) in C_6D_6. 17
Figure S17. ^{13}C NMR spectrum (400 MHz) of parathyrsoidin C (**3**) in C_6D_6. 18
Figure S18. COSY spectrum (400 MHz) of parathyrsoidin C (**3**) in C_6D_6. 19
Figure S19. HSQC spectrum (400 MHz) of parathyrsoidin C (**3**) in C_6D_6. 20
Figure S20. HMBC spectrum (400 MHz) of parathyrsoidin C (**3**) in C_6D_6. 21
Figure S21. NOESY spectrum (400 MHz) of parathyrsoidin C (**3**) in C_6D_6. 22
Figure S22. ^{1}H NMR spectrum (400 MHz) of parathyrsoidin D (**4**) in C_6D_6. 23
Figure S23. ^{13}C NMR spectrum (400 MHz) of parathyrsoidin D (**4**) in C_6D_6. 24
Figure S24. COSY spectrum (400 MHz) of parathyrsoidin D (**4**) in C_6D_6. 25
Figure S25. HSQC spectrum (400 MHz) of parathyrsoidin D (**4**) in C_6D_6. 26
Figure S26. HMBC spectrum (400 MHz) of parathyrsoidin D (**4**) in C_6D_6. 27
Figure S27. NOESY spectrum (400 MHz) of parathyrsoidin D (**4**) in C_6D_6. 28

Figure S1. ^{1}H NMR spectrum (300 MHz) of parathyrsoidin A (**1**) in $CDCl_3$.

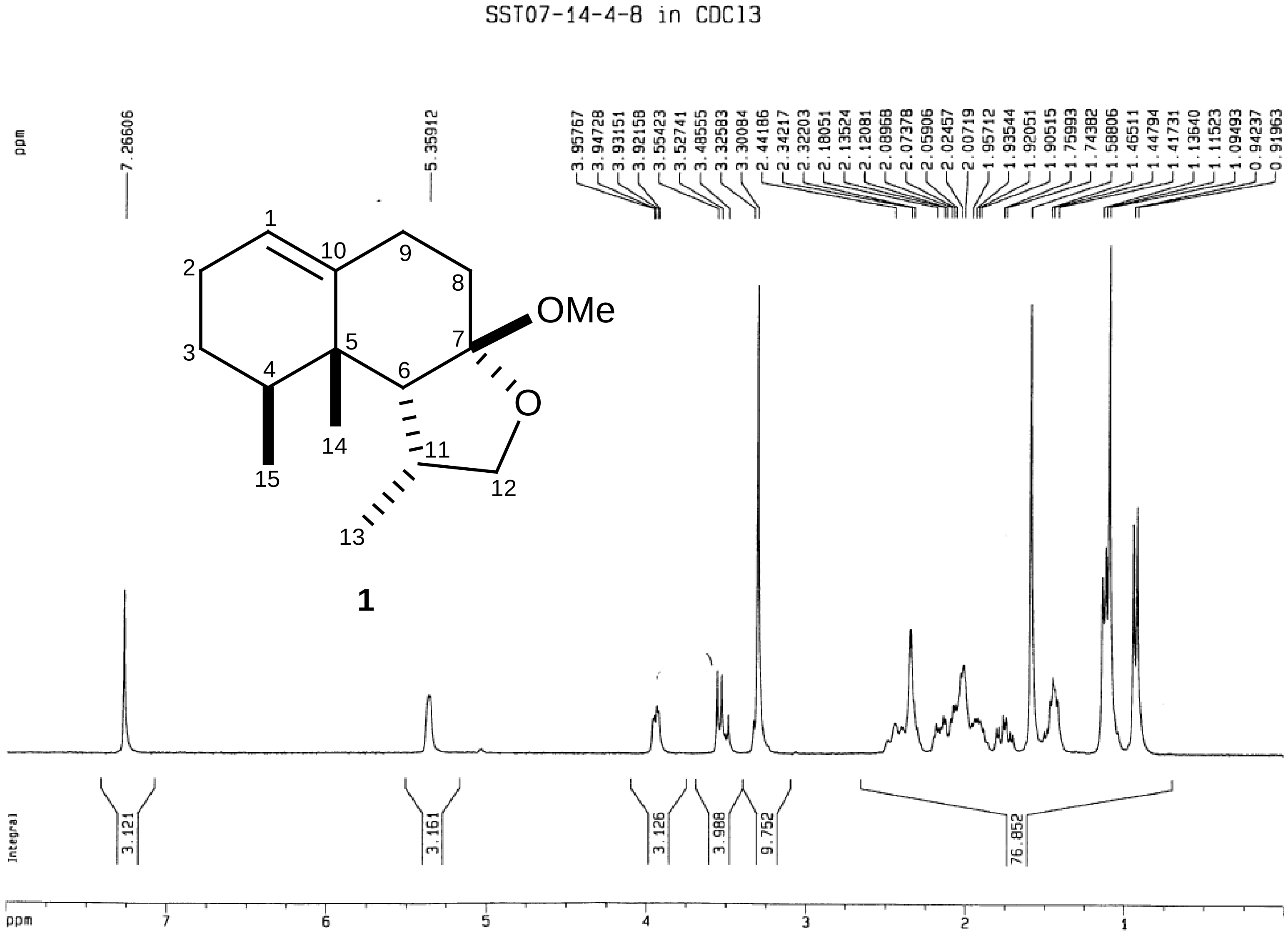

Figure S2. ^{1}H NMR spectrum (400 MHz) of parathyrsoidin A (**1**) in C_6D_6.

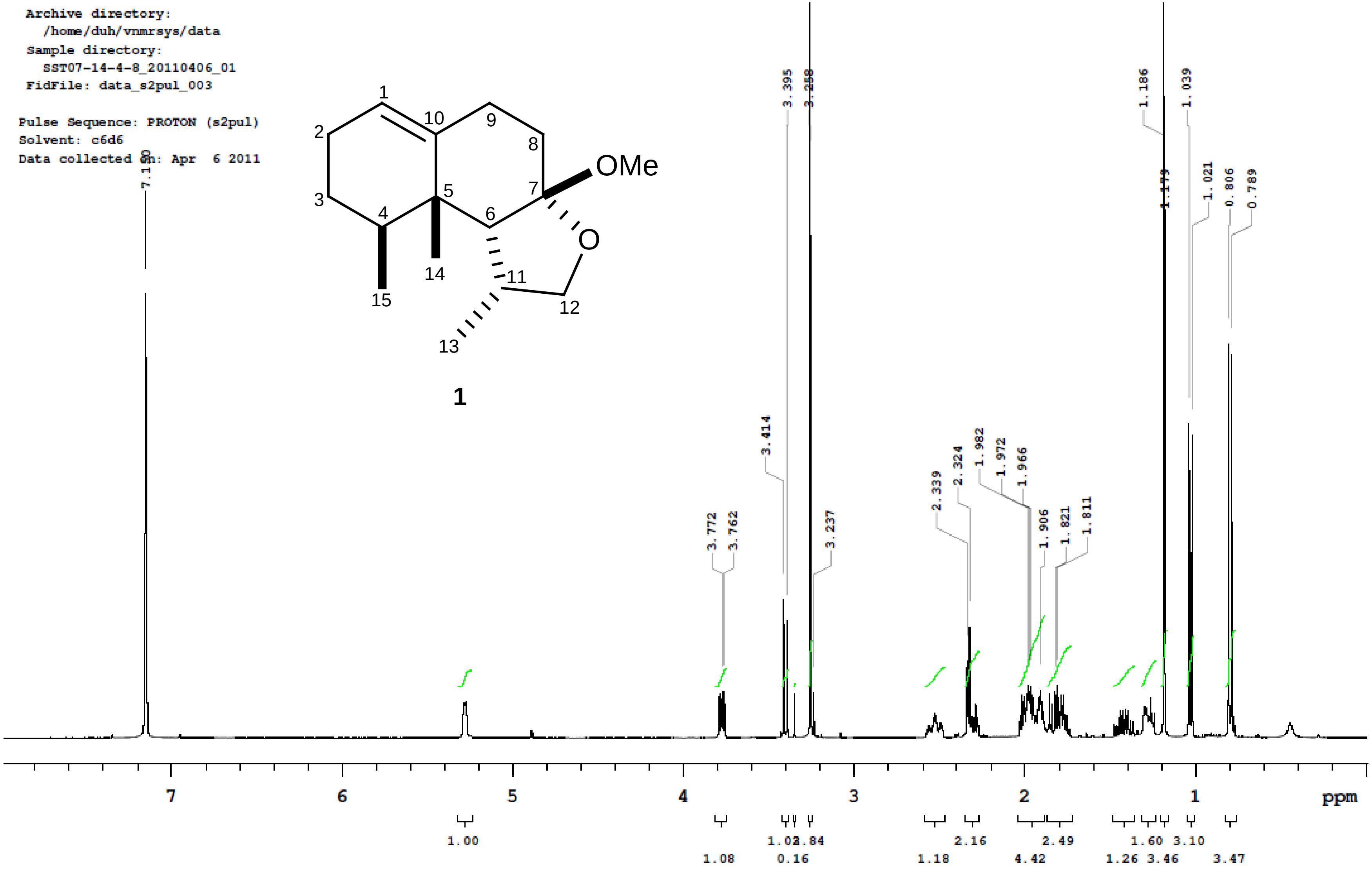

Mar. Drugs **2013**, *11*

Figure S3. ^{13}C NMR spectrum (400 MHz) of parathyrsoidin A (**1**) in C_6D_6.

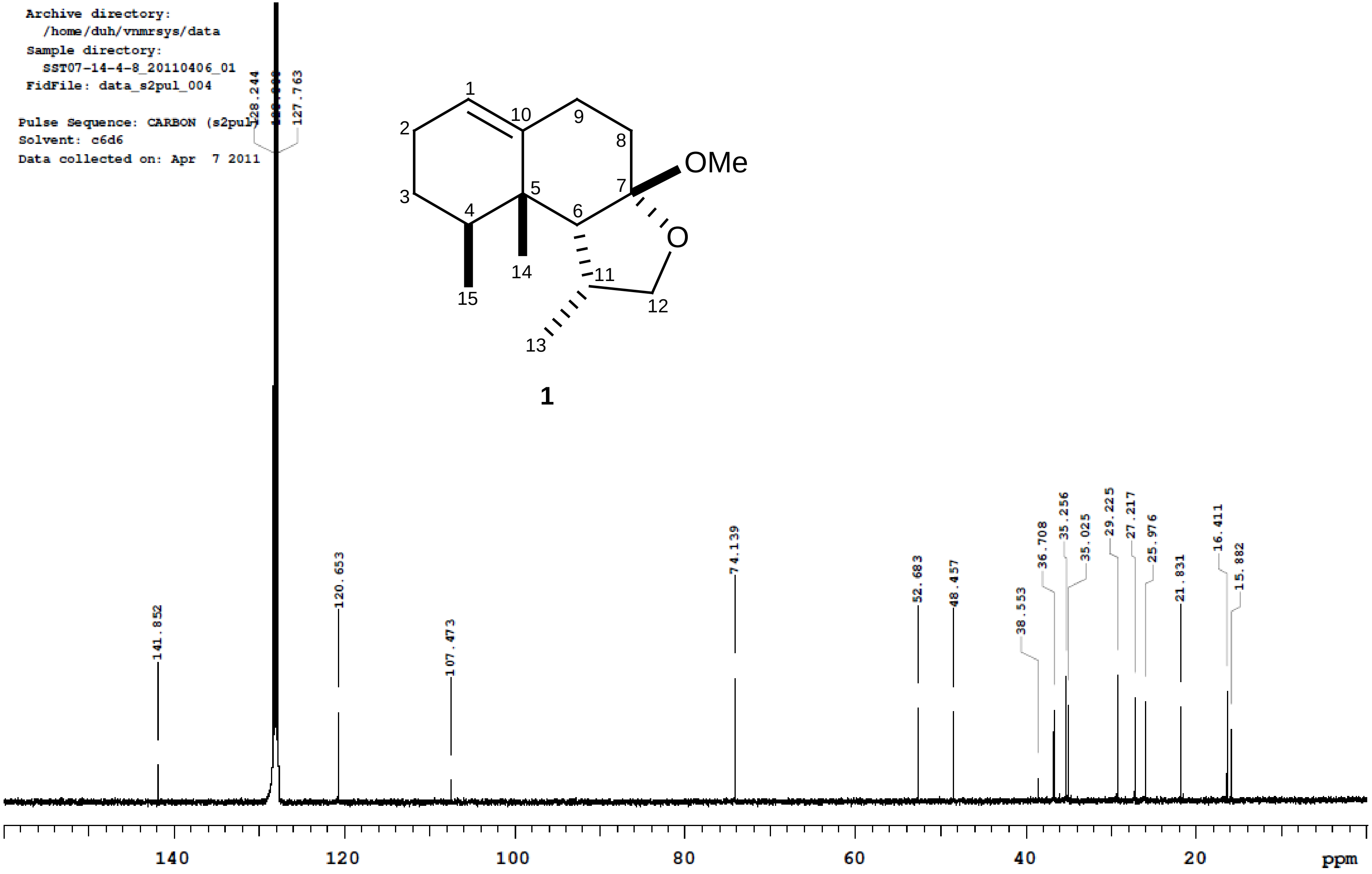

Mar. Drugs **2013**, *11*

Figure S4. COSY spectrum (400 MHz) of parathyrsoidin A (**1**) in C_6D_6.

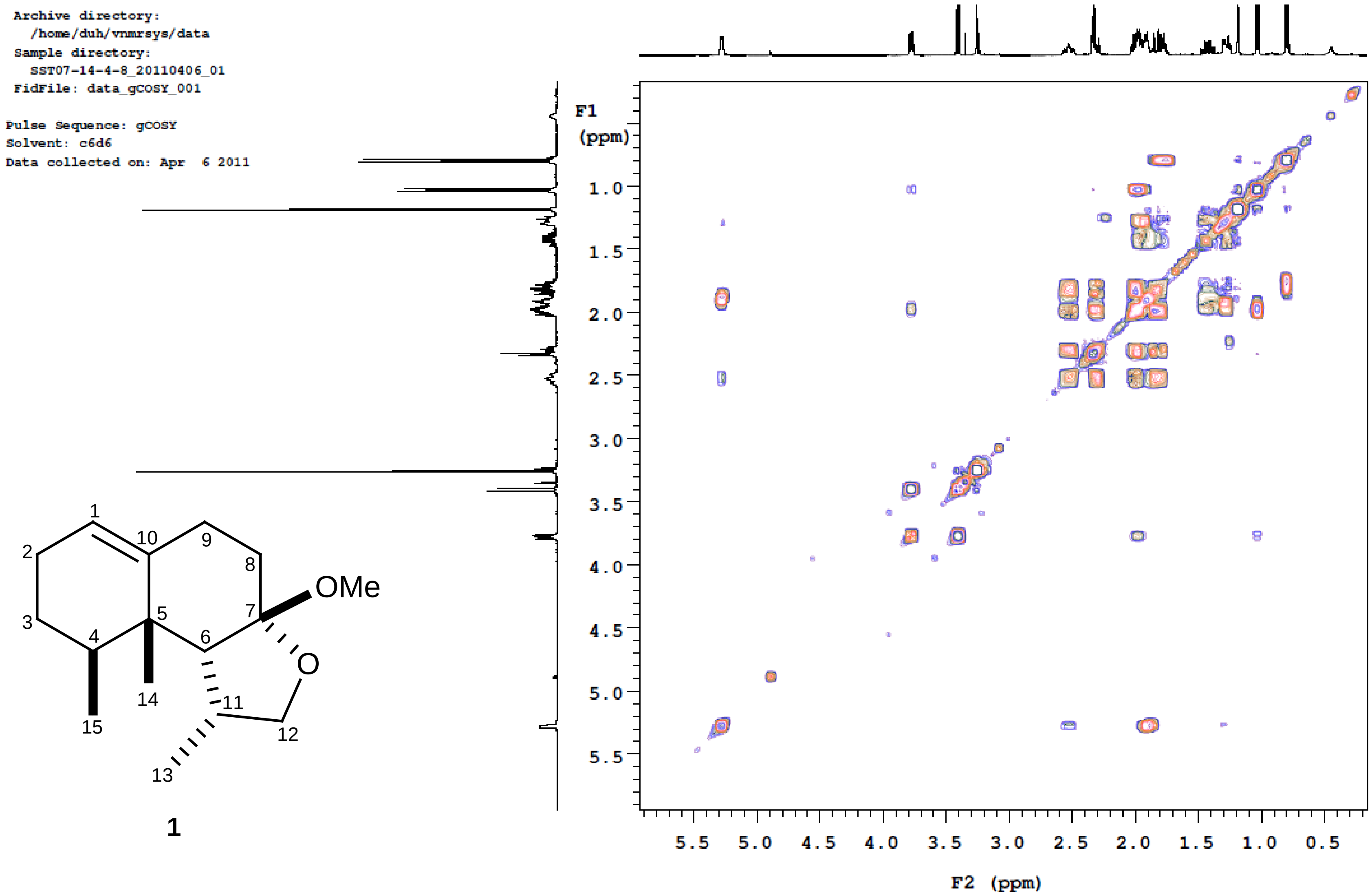

Mar. Drugs **2013**, *11*

Figure S5. HSQC spectrum (400 MHz) of parathyrsoidin A (**1**) in C_6D_6.

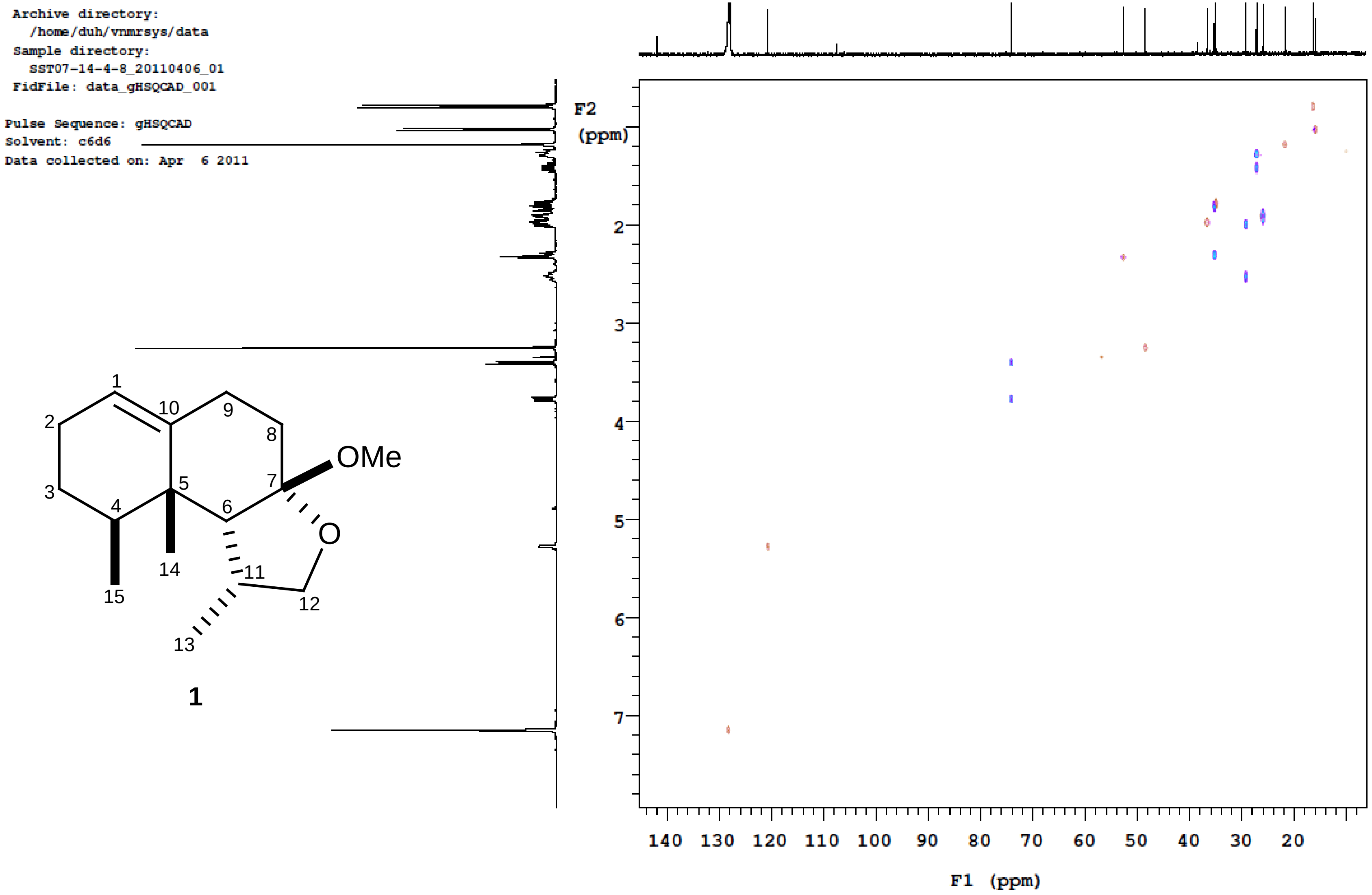

Figure S6. HMBC spectrum (400 MHz) of parathyrsoidin A (**1**) in C_6D_6.

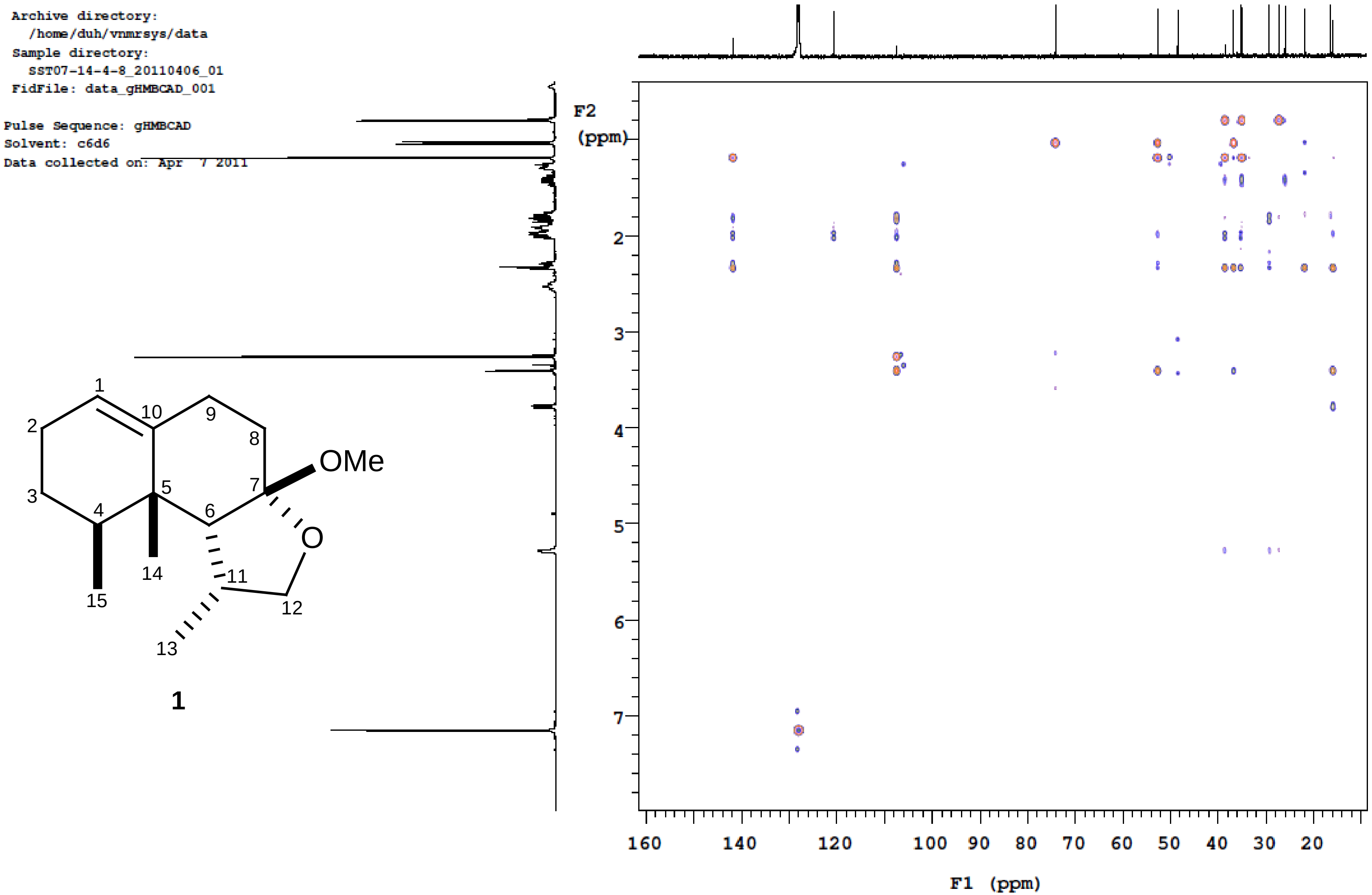

Figure S7. NOESY spectrum (400 MHz) of parathyrsoidin A (**1**) in C_6D_6.

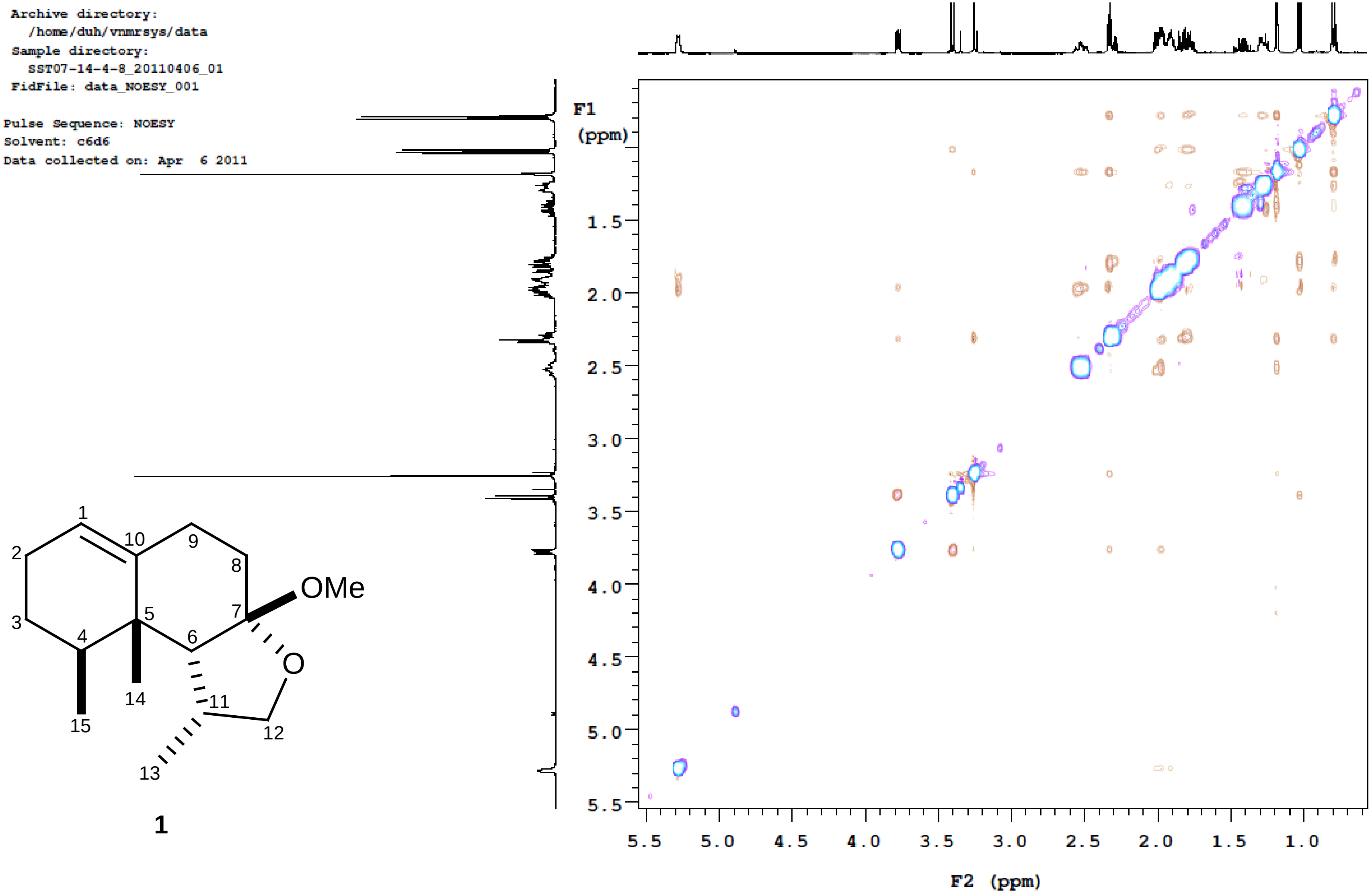

1

Figure S8. ^{1}H NMR spectrum (300 MHz) of parathyrsoidin B (**2**) in $CDCl_3$.

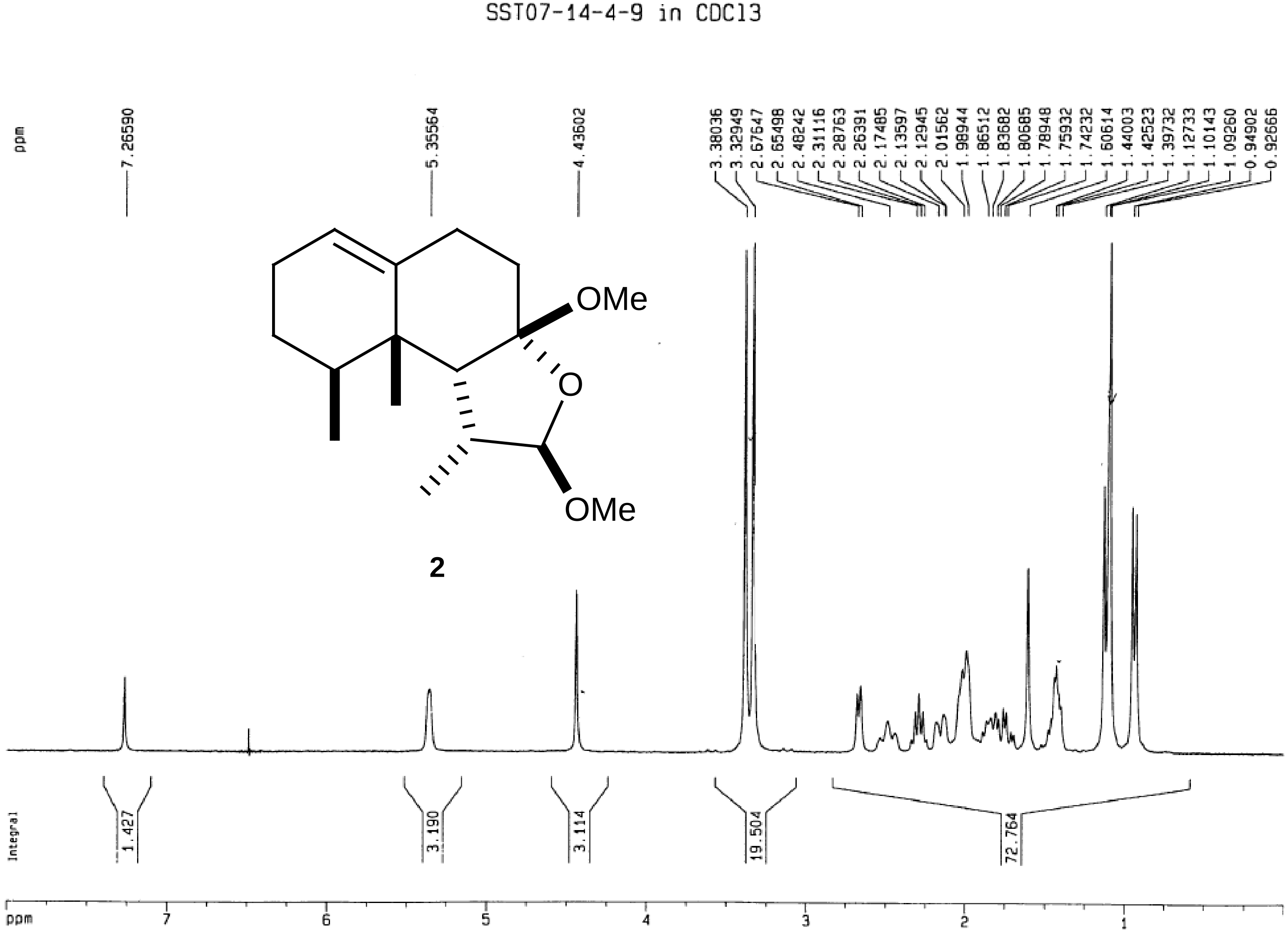

Mar. Drugs **2013**, *11*

Figure S9. ^{1}H NMR spectrum (400 MHz) of parathyrsoidin B (**2**) in C_6D_6.

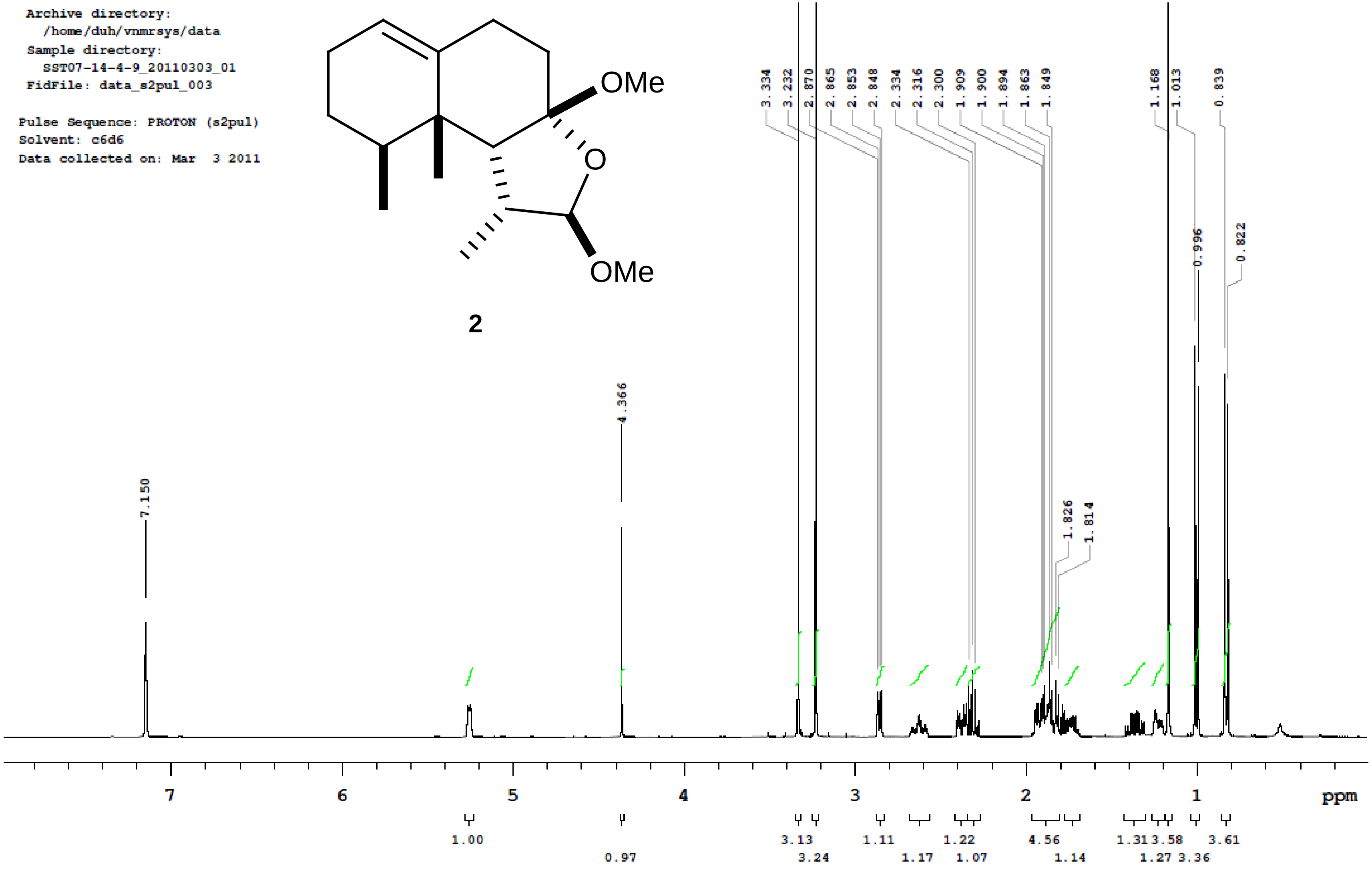

Mar. Drugs **2013**, *11*

Figure S10. ^{13}C NMR spectrum (400 MHz) of parathyrsoidin B (**2**) in C_6D_6.

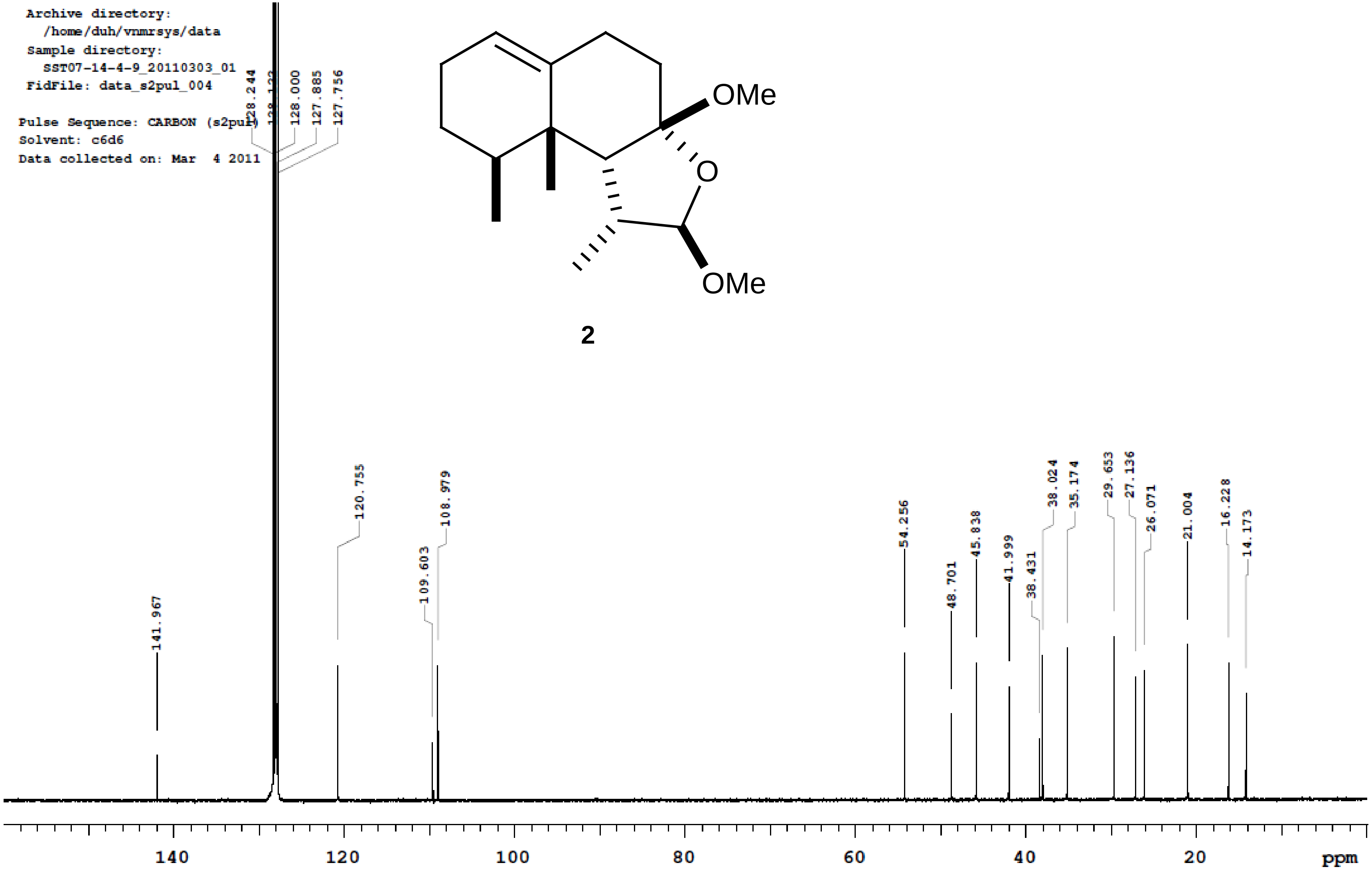

Mar. Drugs **2013**, *11*

Figure S11. COSY spectrum (400 MHz) of parathyrsoidin B (**2**) in C_6D_6.

Archive directory:
/home/duh/vnmrsys/data
Sample directory:
SST07-14-4-9_20110303_01
FidFile: data_gCOSY_001

Pulse Sequence: gCOSY
Solvent: c6d6
Data collected on: Mar 3 2011

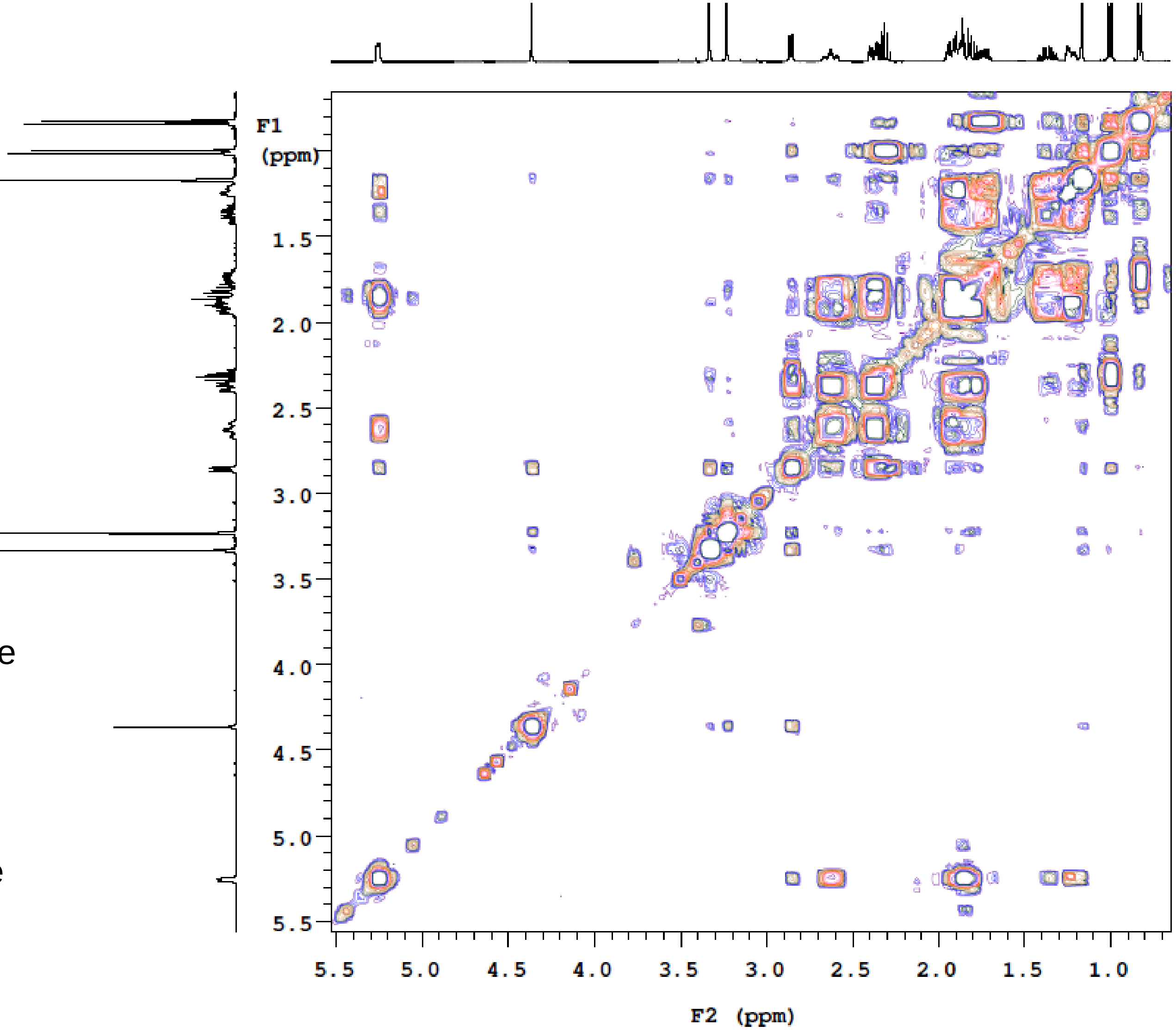

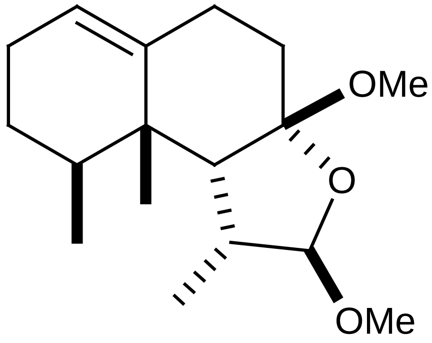

2

Mar. Drugs **2013**, *11*

Figure S12. HSQC spectrum (400 MHz) of parathyrsoidin B (**2**) in C_6D_6.

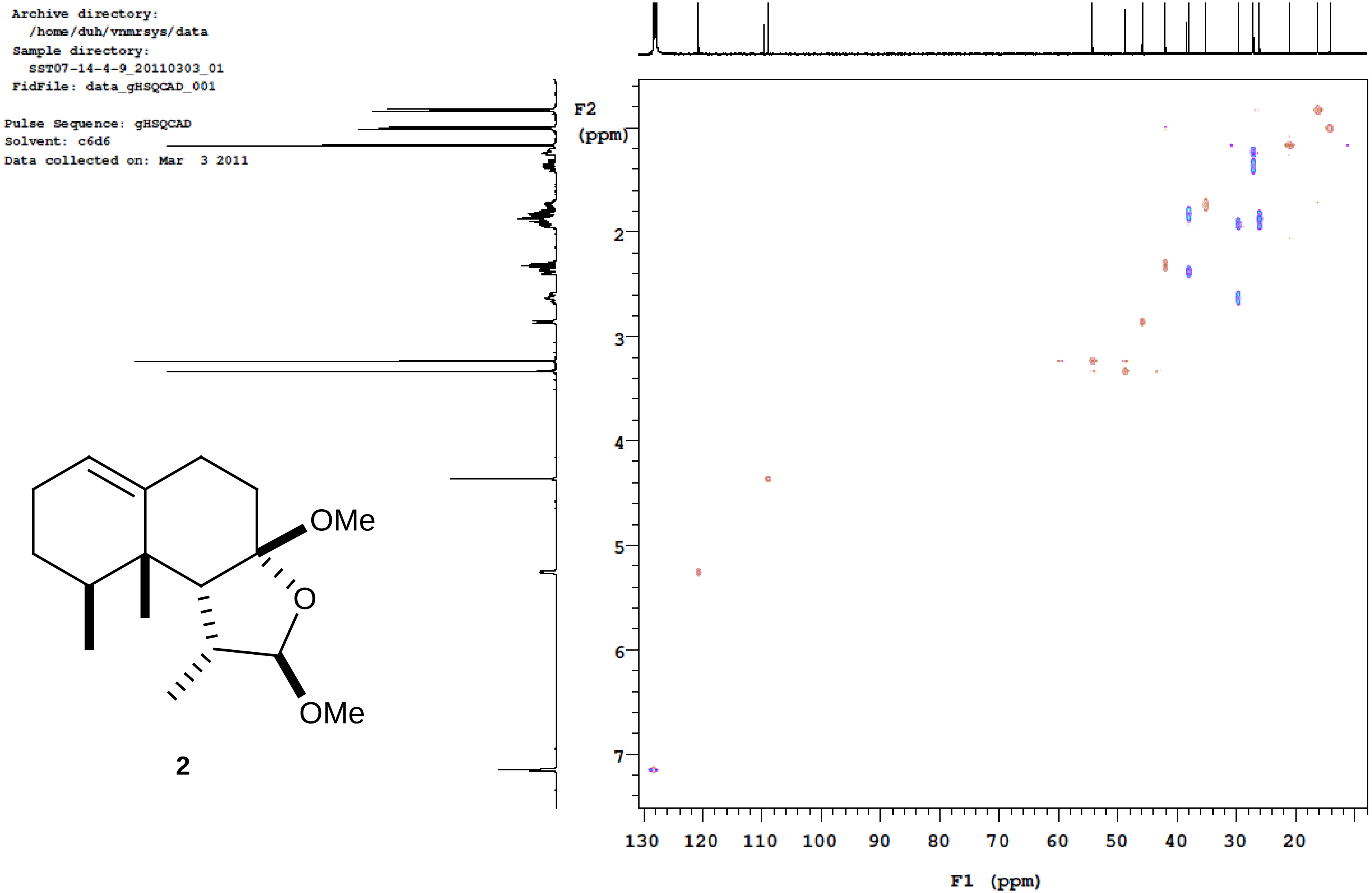

Mar. Drugs **2013**, *11*

Figure S13. HMBC spectrum (400 MHz) of parathyrsoidin B (**2**) in C_6D_6.

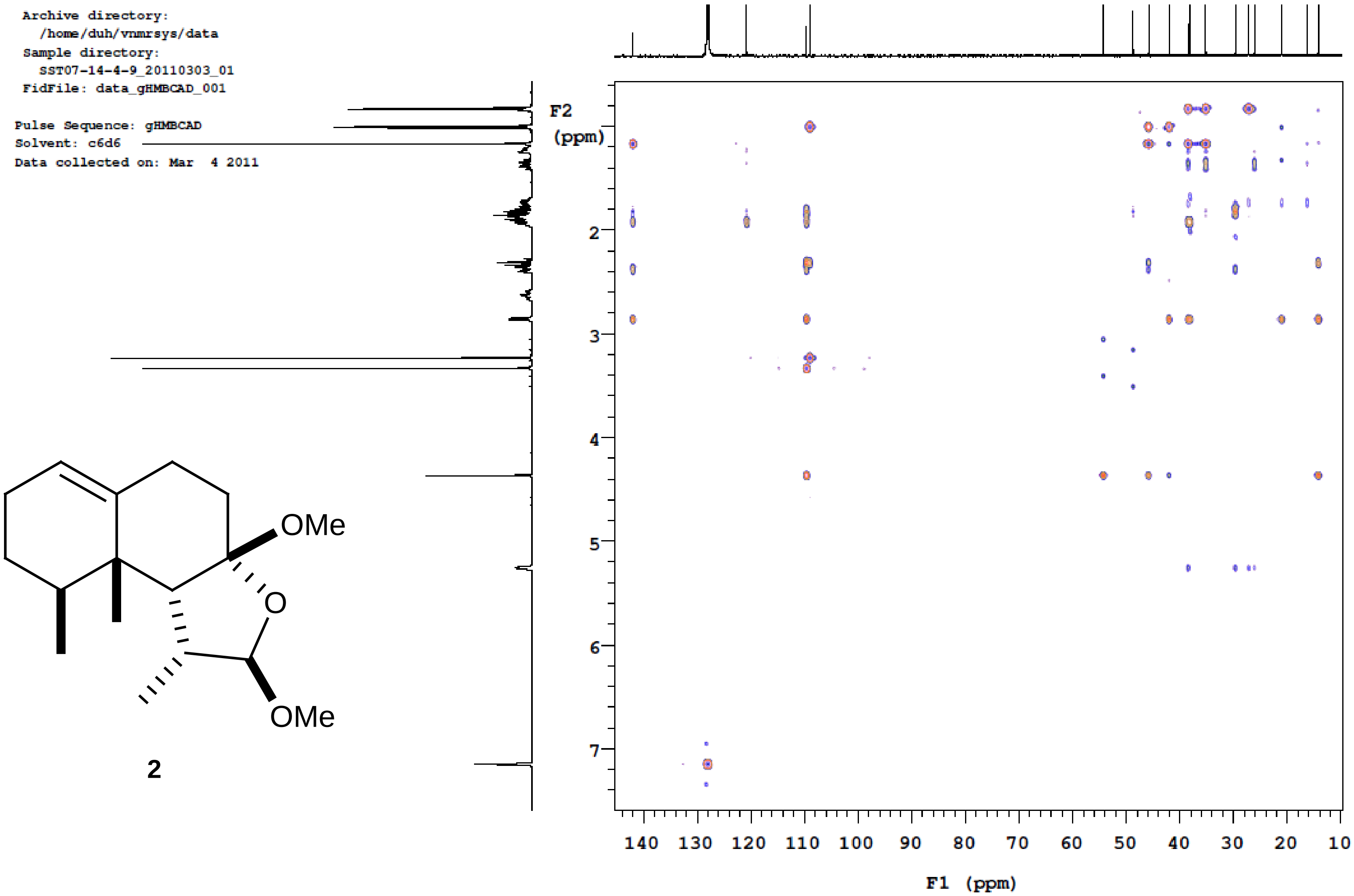

Figure S14. NOESY spectrum (400 MHz) of parathyrsoidin B (**2**) in C_6D_6.

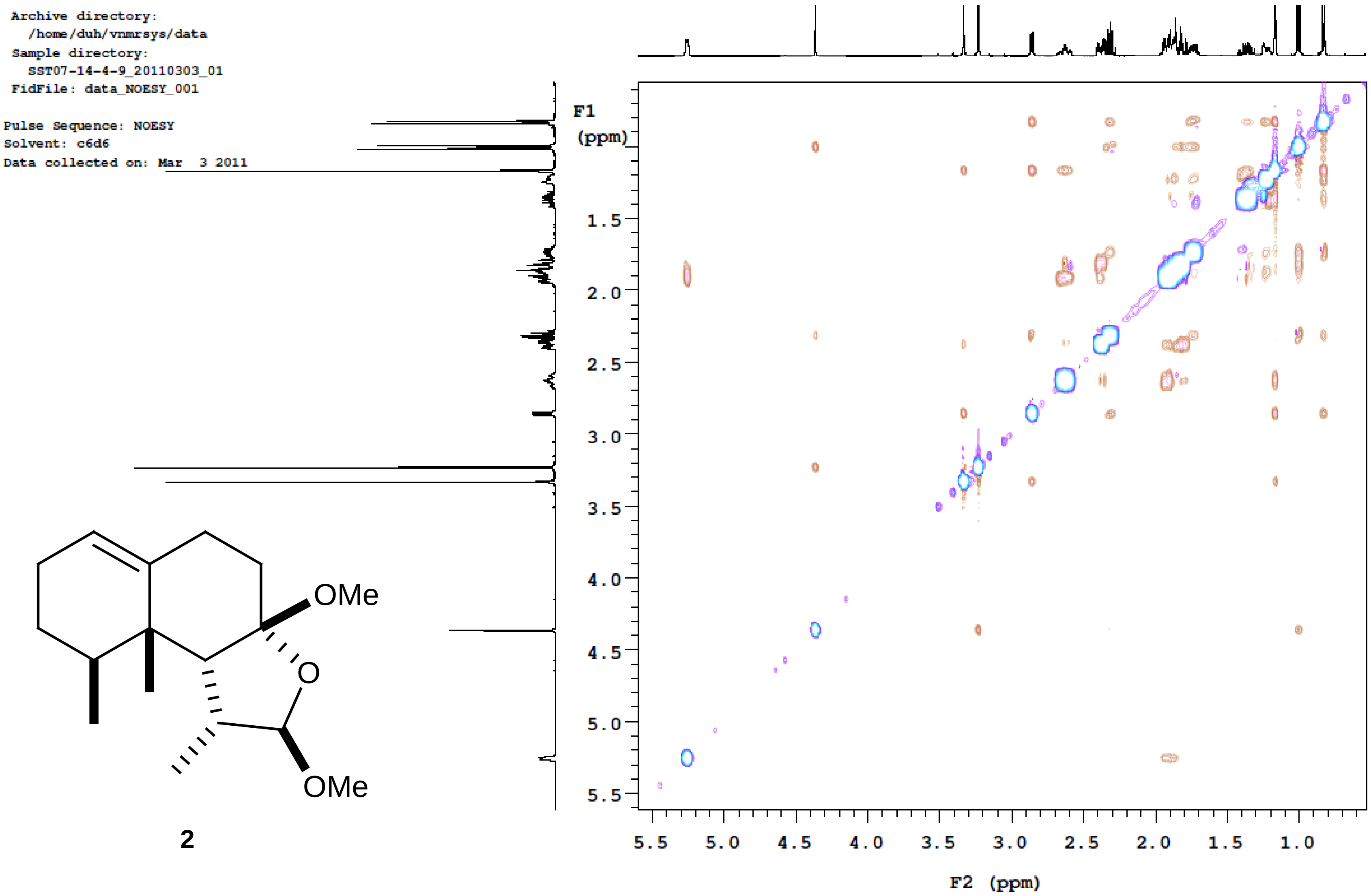

Figure S15. ^{1}H NMR spectrum (300 MHz) of parathyrsoidin C (**3**) in $CDCl_3$.

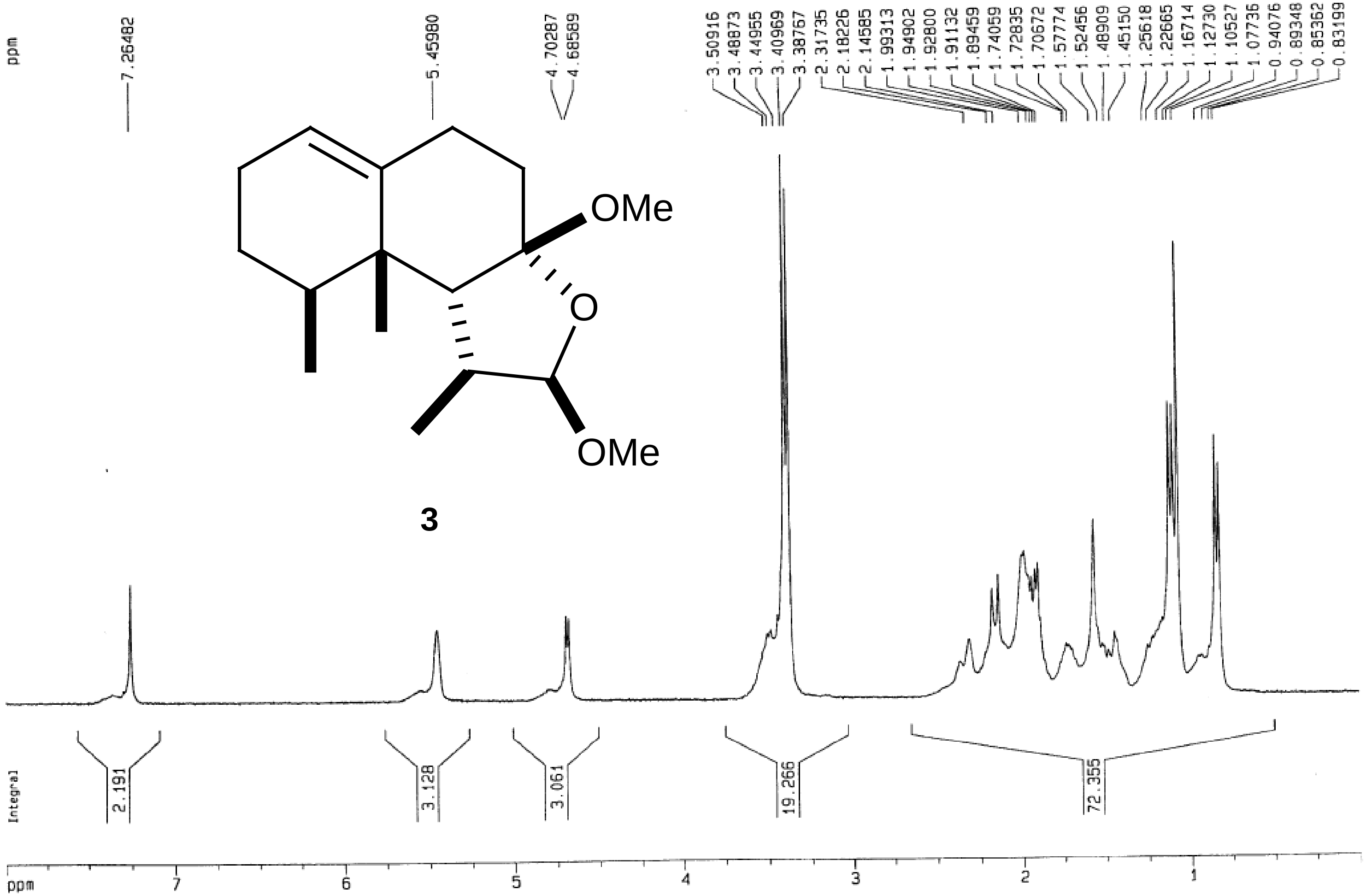

Mar. Drugs **2013**, *11*

Figure S16. ^{1}H NMR spectrum (400 MHz) of parathyrsoidin C (**3**) in C_6D_6.

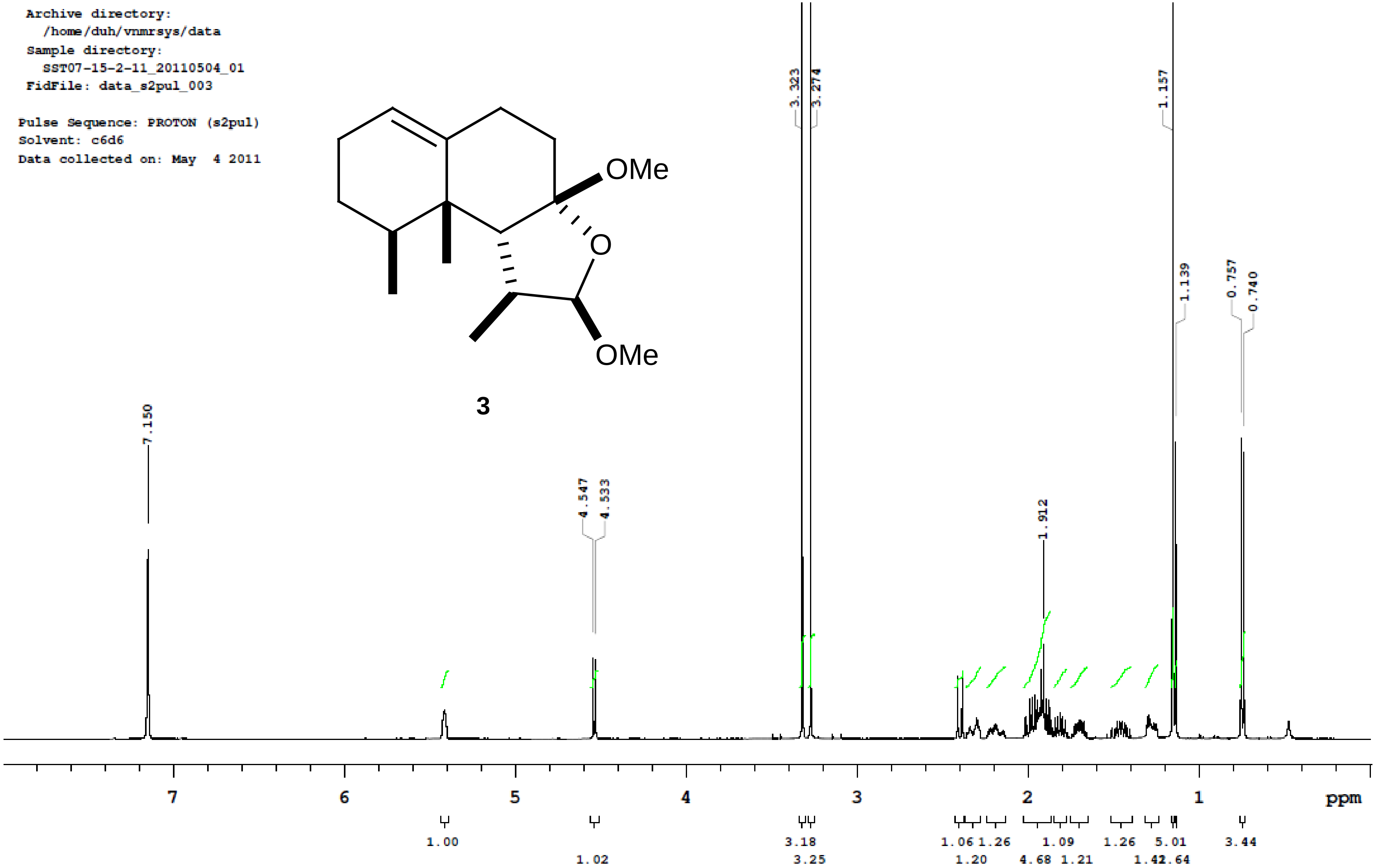

Mar. Drugs **2013**, *11*

Figure S17. ^{13}C NMR spectrum (400 MHz) of parathyrsoidin C (**3**) in C_6D_6.

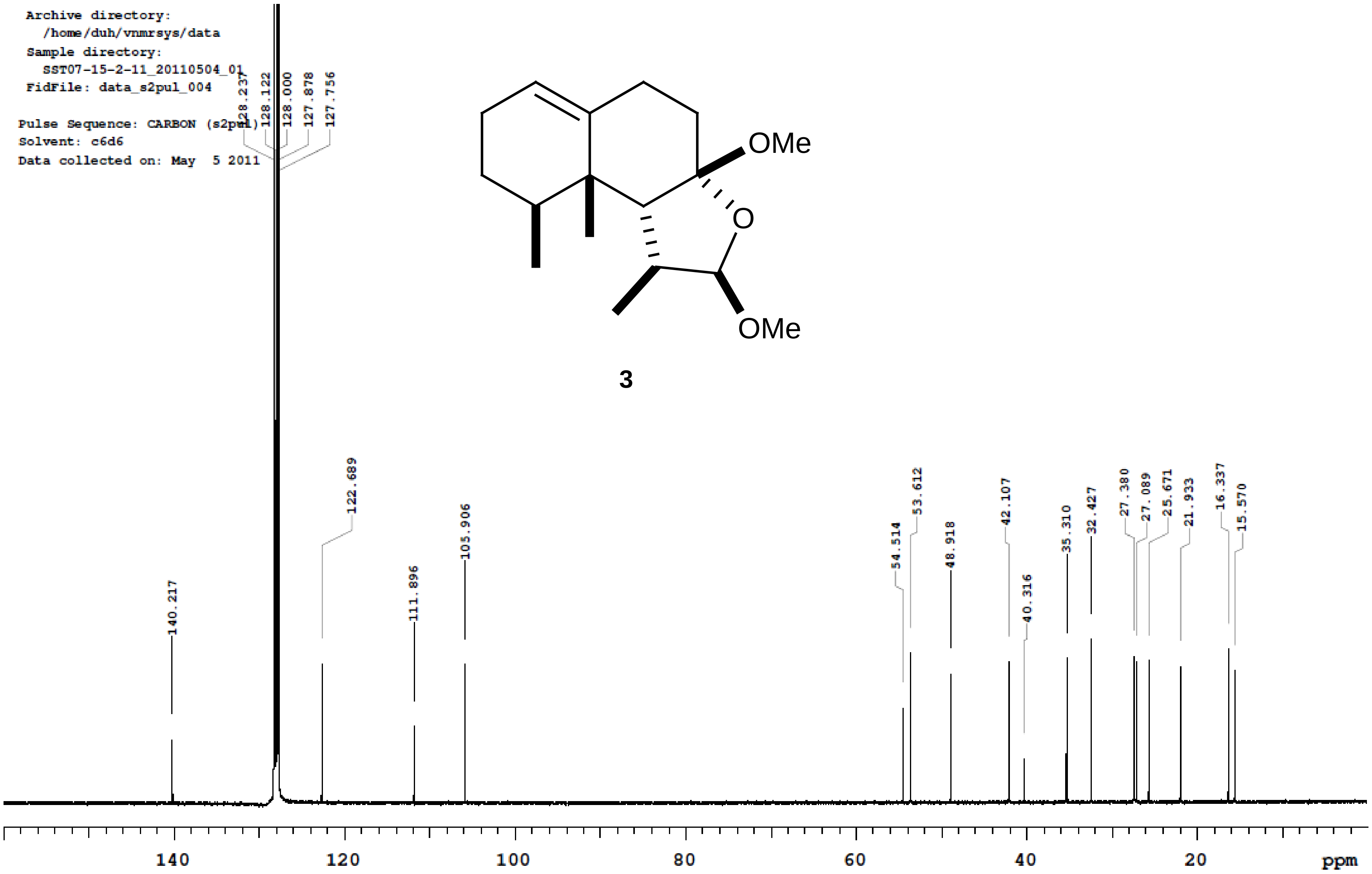

Mar. Drugs **2013**, *11*

Figure S18. COSY spectrum (400 MHz) of parathyrsoidin C (**3**) in C_6D_6.

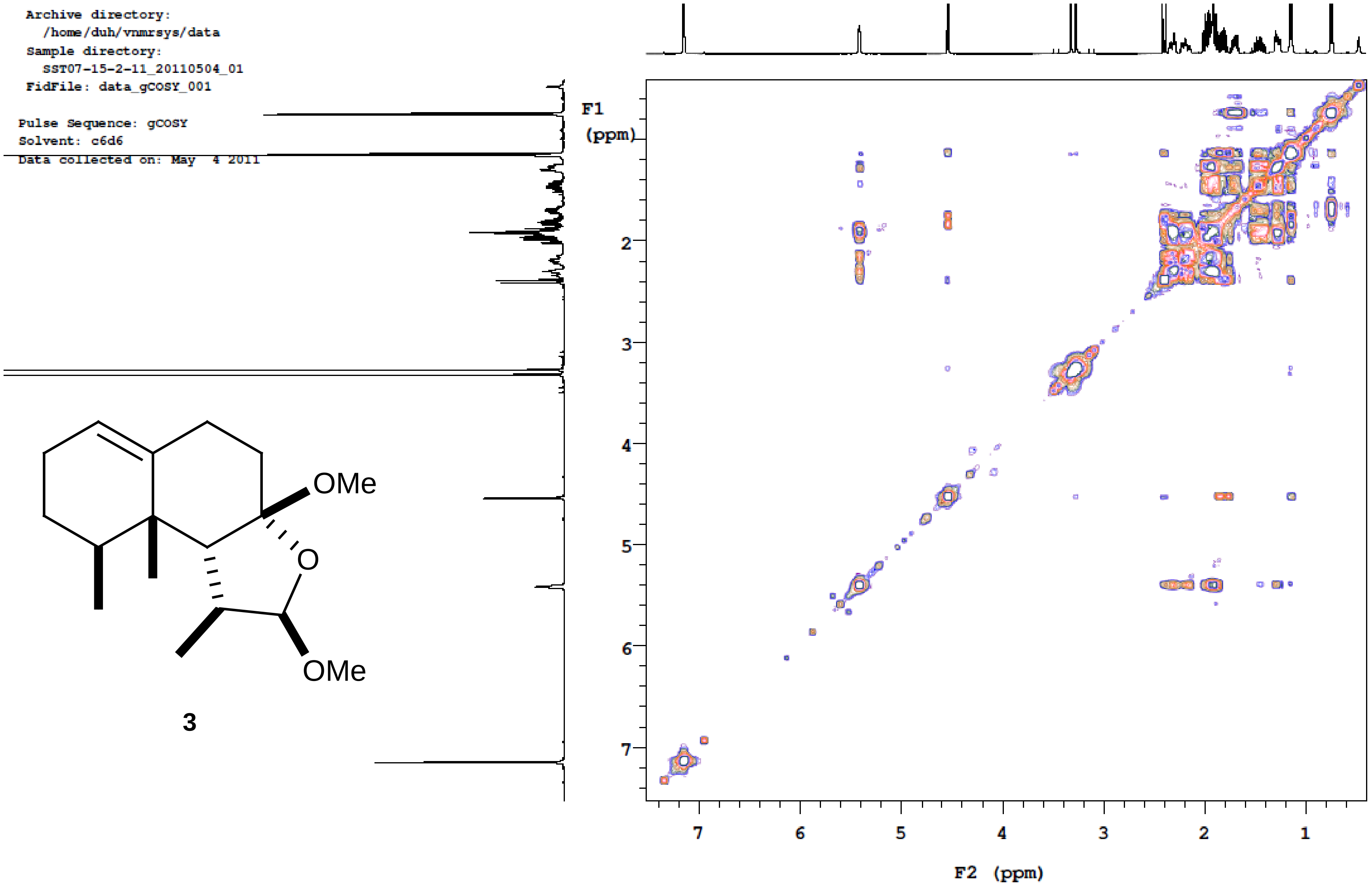

Mar. Drugs **2013**, *11*

Figure S19. HSQC spectrum (400 MHz) of parathyrsoidin C (**3**) in C_6D_6.

SST07-15-2-11

Probe: dual
Pulse Sequence: gHSQCAD

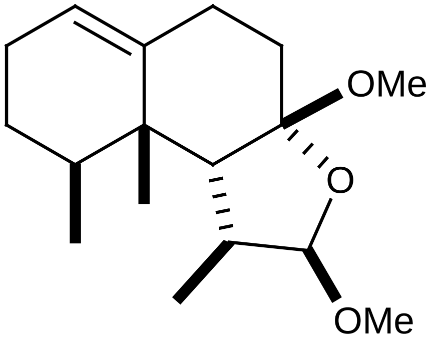

3

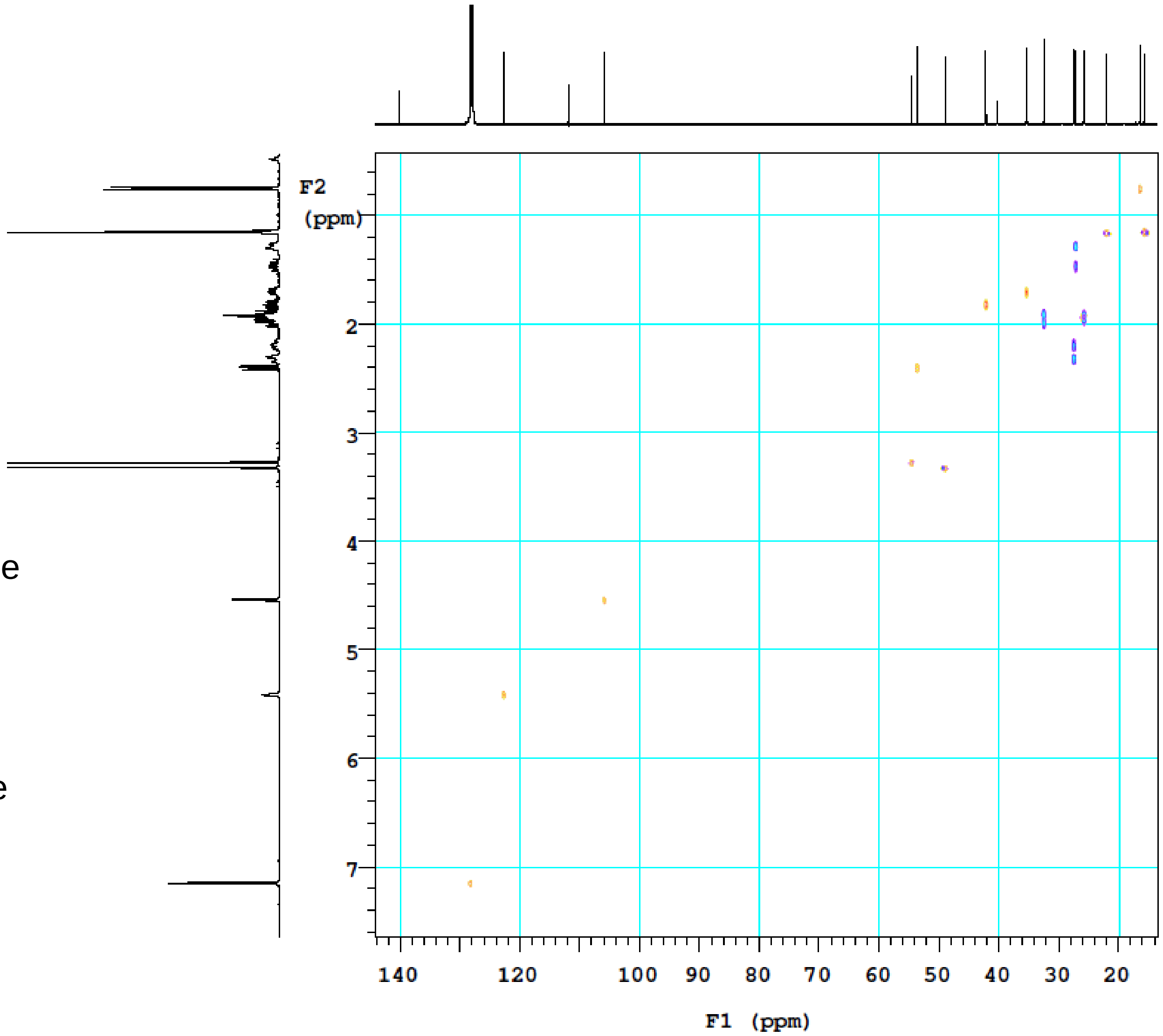

Mar. Drugs **2013**, *11*

Figure S20. HMBC spectrum (400 MHz) of parathyrsoidin C (**3**) in C_6D_6.

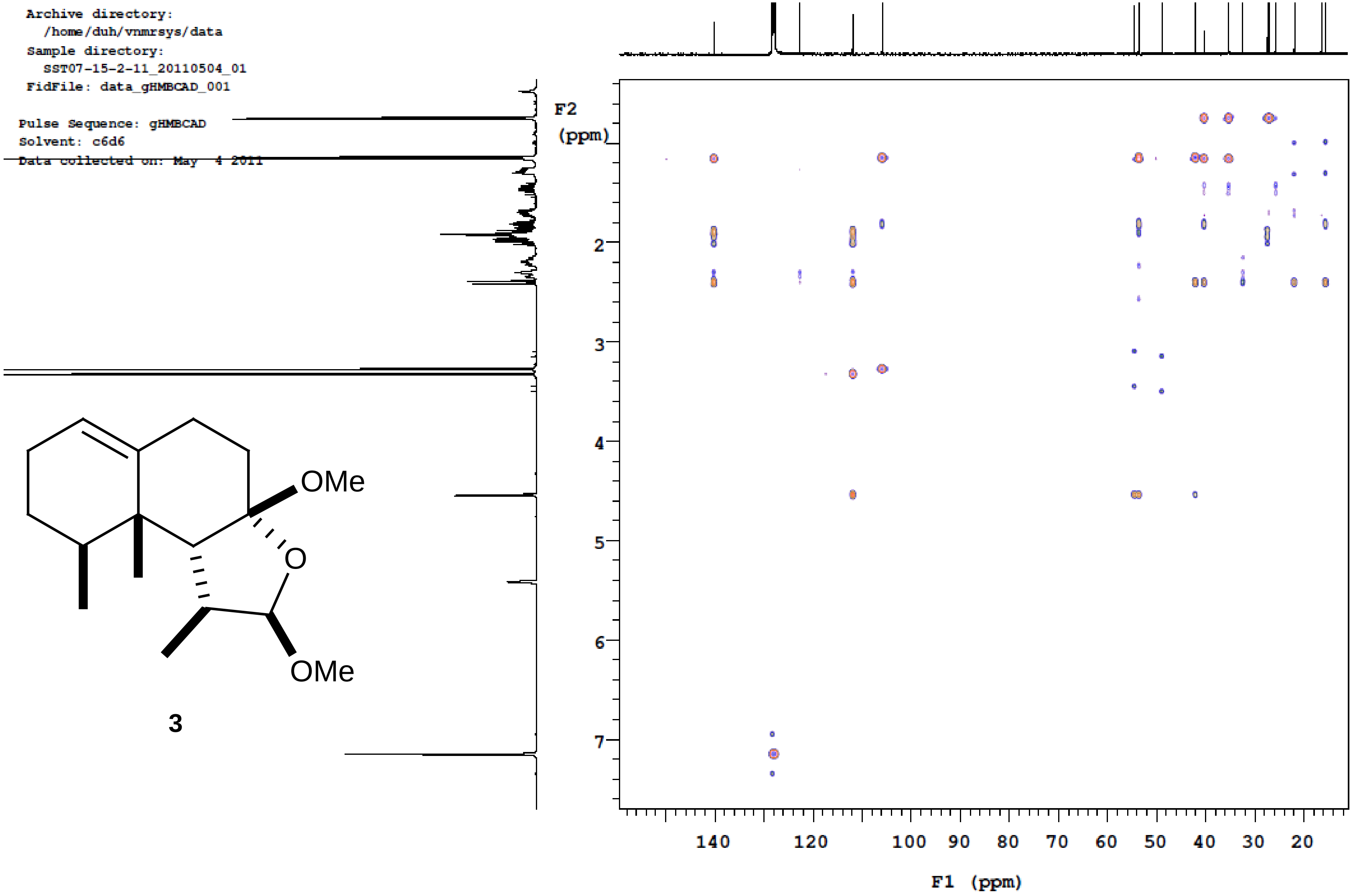

Figure S21. NOESY spectrum (400 MHz) of parathyrsoidin C (**3**) in C_6D_6.

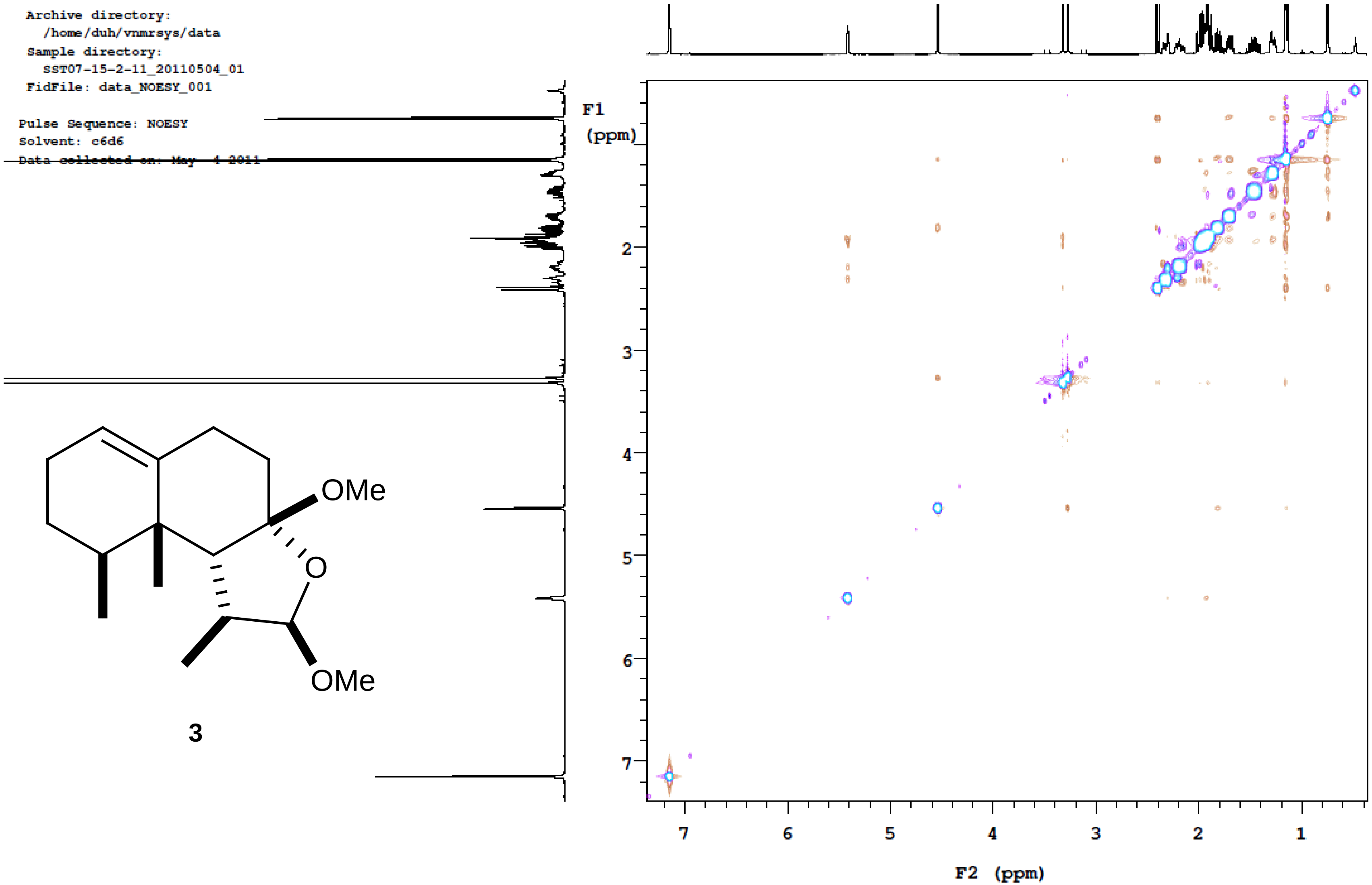

3

Figure S22. ^{1}H NMR spectrum (400 MHz) of parathyrsoidin D (**4**) in C_6D_6.

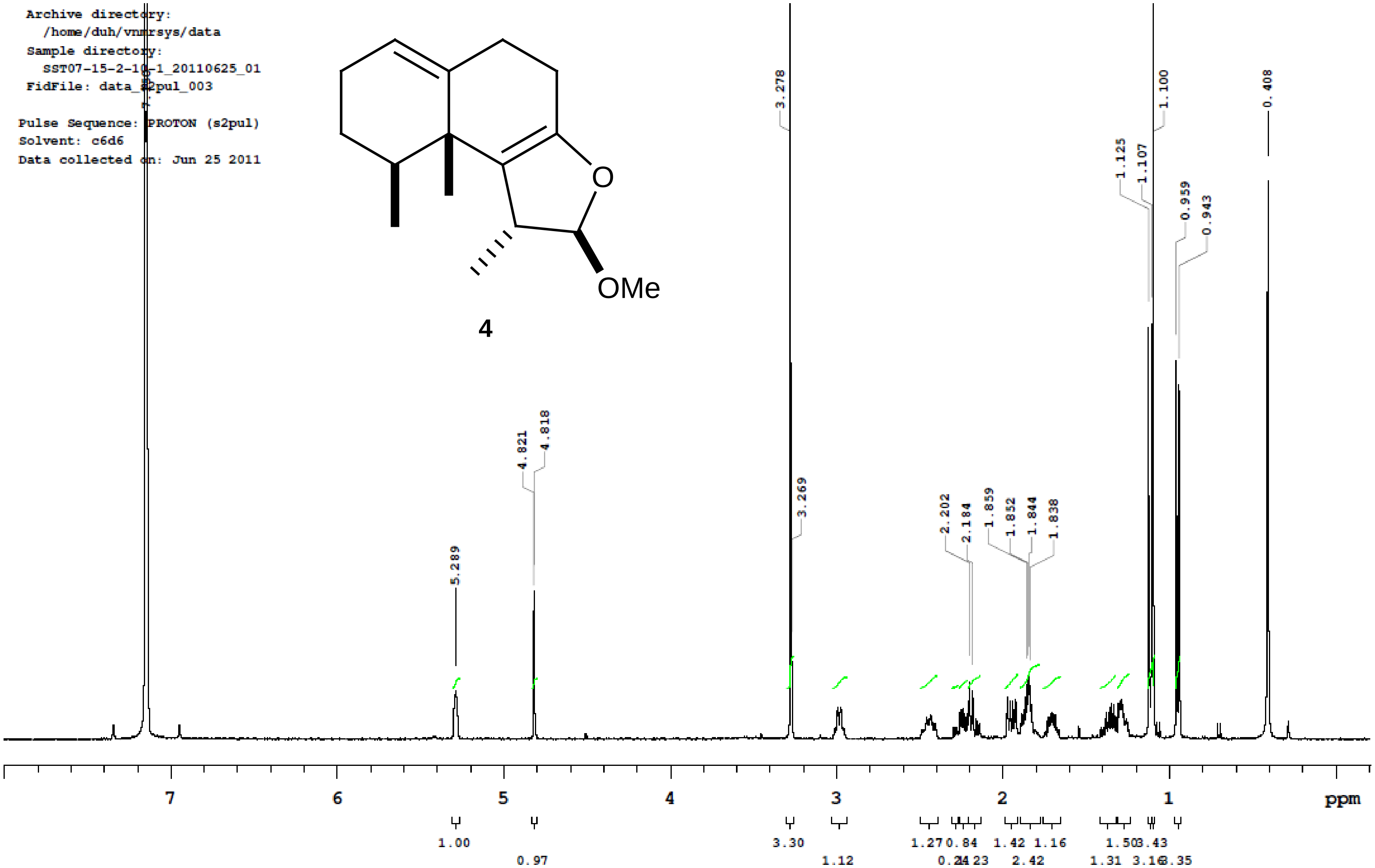

Mar. Drugs **2013**, *11*

Figure S23. ^{13}C NMR spectrum (400 MHz) of parathyrsoidin D (**4**) in C_6D_6.

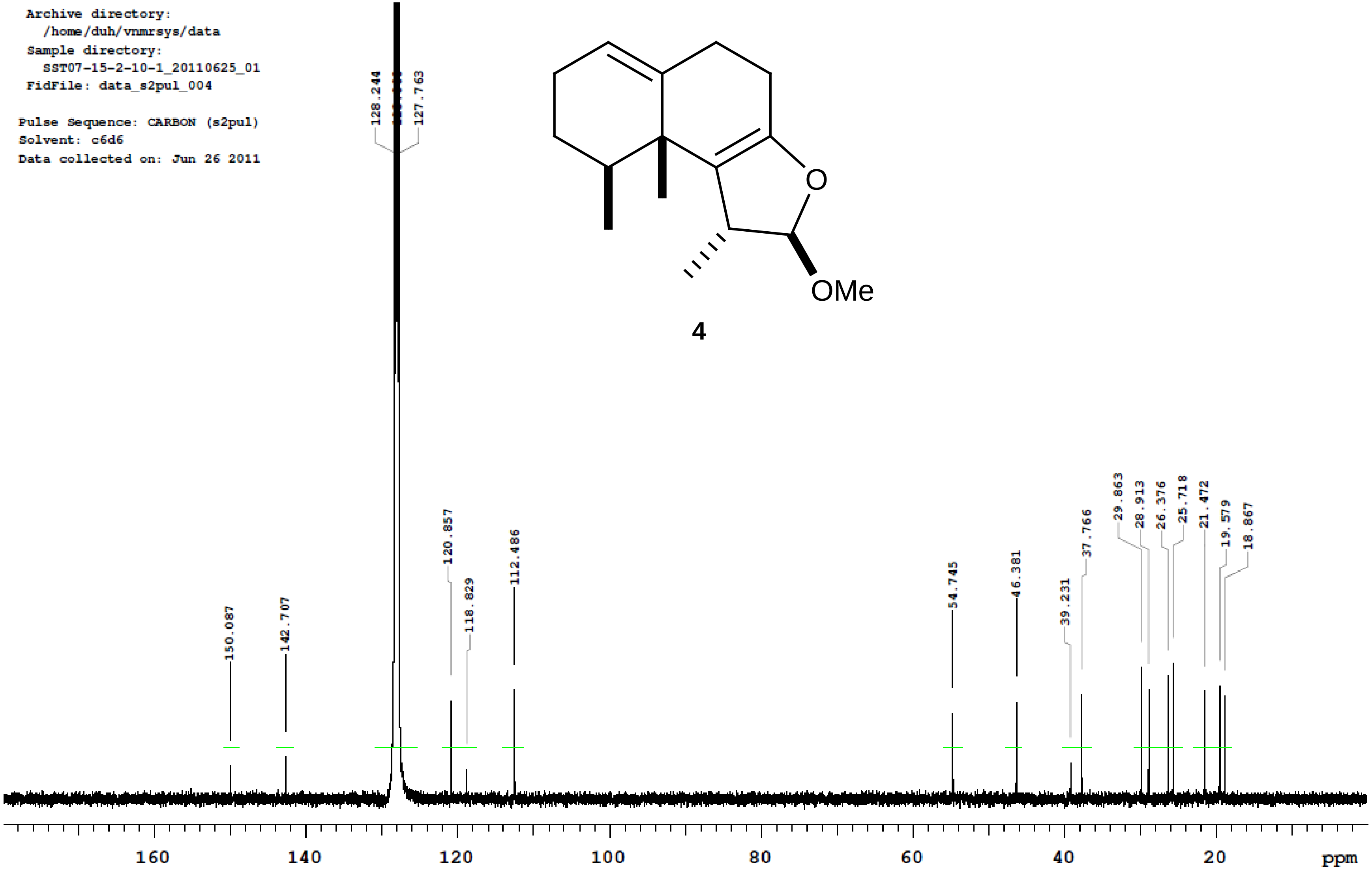

Mar. Drugs **2013**, *11*

Figure S24. COSY spectrum (400 MHz) of parathyrsoidin D (**4**) in C_6D_6.

Archive directory:
/home/duh/vnmrsys/data
Sample directory:
SST07-15-2-10-1_20110625_01
FidFile: data_gCOSY_001

Pulse Sequence: gCOSY
Solvent: c6d6
Data collected on: Jun 25 2011

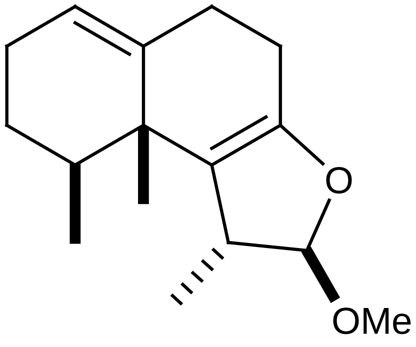

4

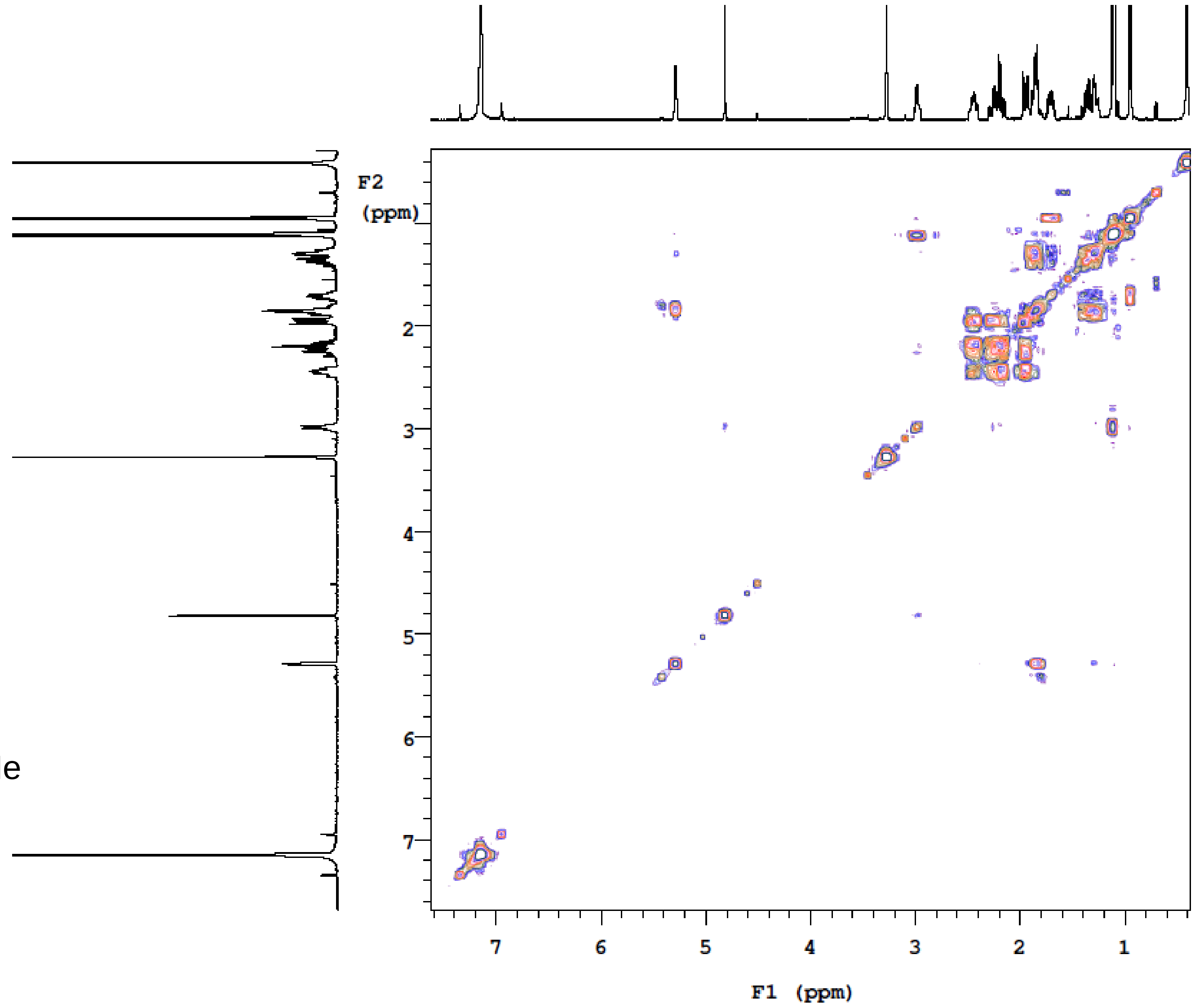

Mar. Drugs **2013**, *11*

Figure S25. HSQC spectrum (400 MHz) of parathyrsoidin D (**4**) in C_6D_6.

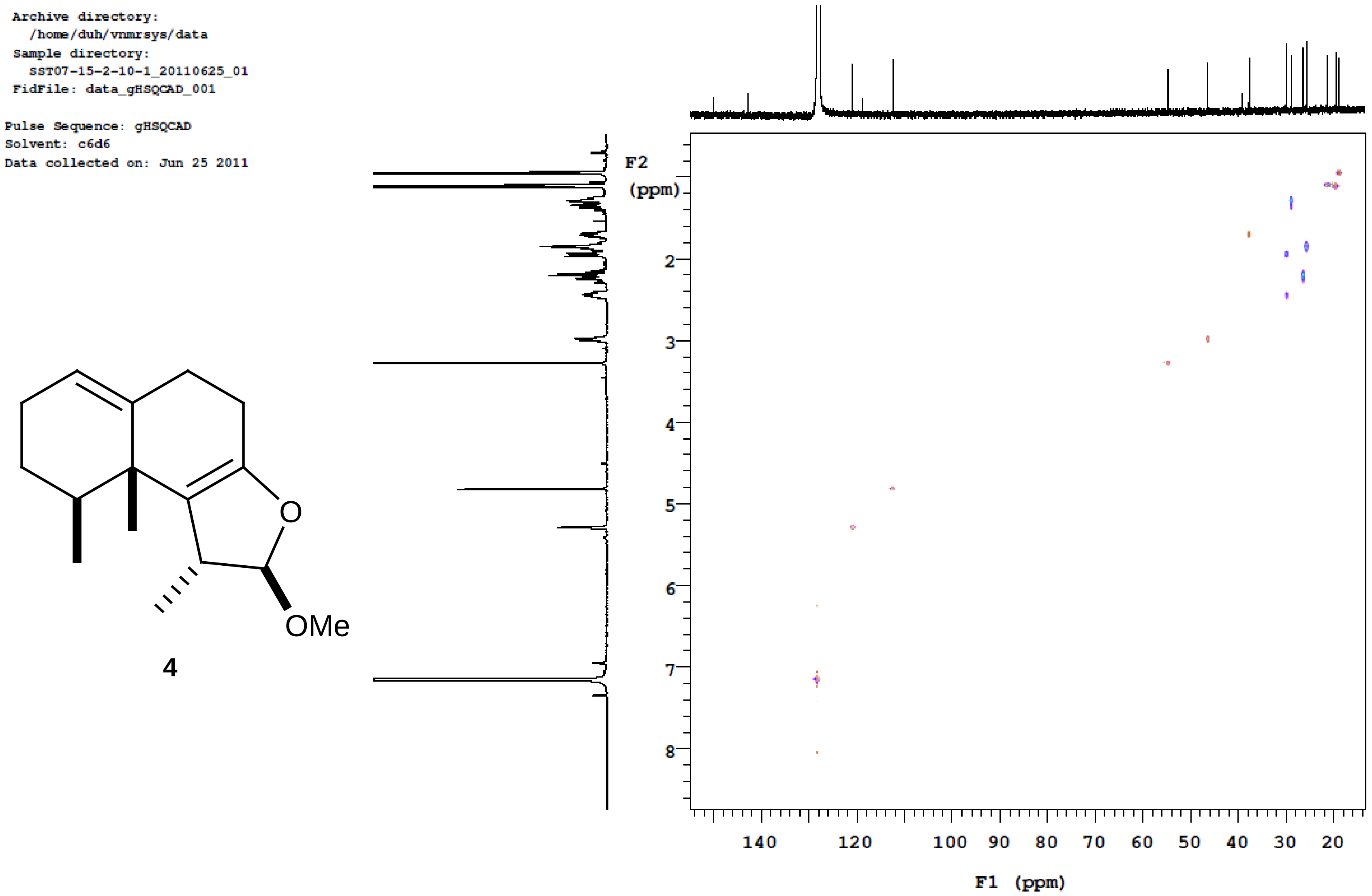

Mar. Drugs **2013**, *11*

Figure S26. HMBC spectrum (400 MHz) of parathyrsoidin D (**4**) in C_6D_6.

Archive directory:
/home/duh/vnmrsys/data
Sample directory:
SST07-15-2-10-1_20110625_01
FidFile: data_gHMBCAD_001

Pulse Sequence: gHMBCAD
Solvent: c6d6
Data collected on: Jun 26 2011

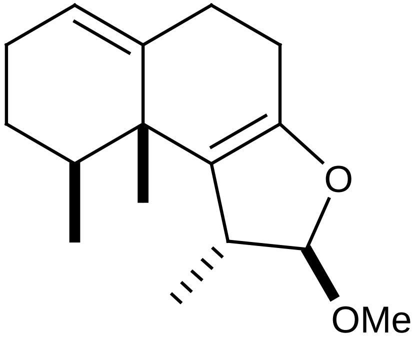

4

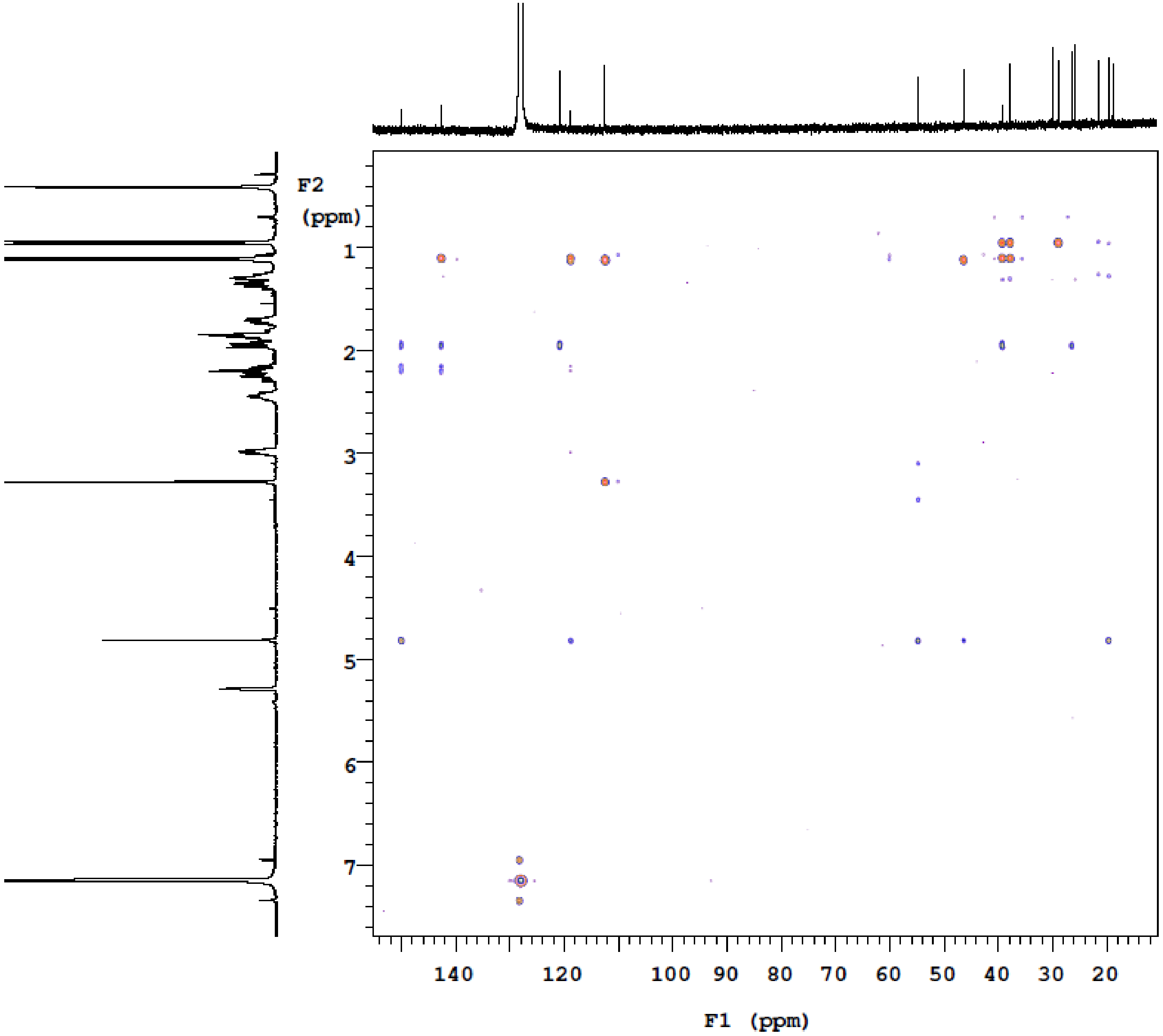

Mar. Drugs **2013**, *11*

Figure S27. NOESY spectrum (400 MHz) of parathyrsoidin D (**4**) in C_6D_6.

Archive directory:
/home/duh/vnmrsys/data
Sample directory:
SST07-15-2-10-1_20110625_01
FidFile: data_NOESY_001

Pulse Sequence: NOESY
Solvent: c6d6
Data collected on: Jun 25 2011

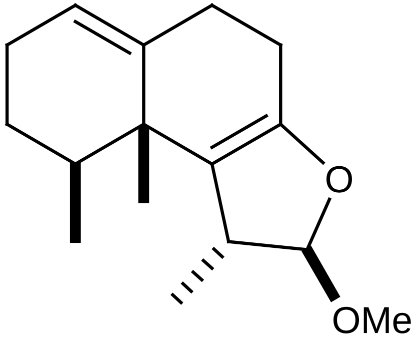

4

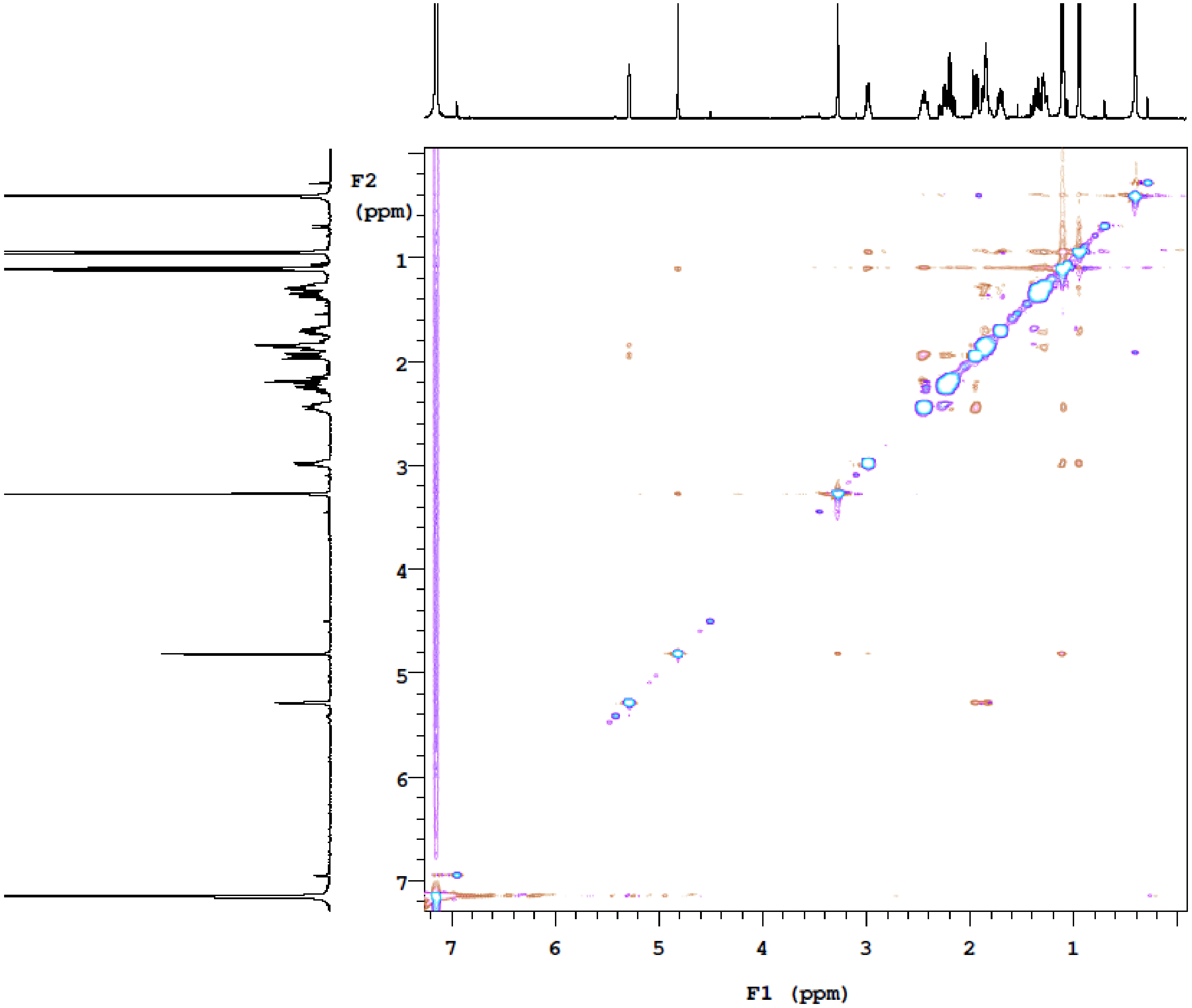

Supplement: Supplementary File 1 — Supplementary Materials (PDF, 1319 KB) [file marinedrugs-11-02501-s001.pdf]
